# Pharmaceuticals and Personal Care Products in the Environment: What Are the Big Questions?

**DOI:** 10.1289/ehp.1104477

**Published:** 2012-05-30

**Authors:** Alistair B.A. Boxall, Murray A. Rudd, Bryan W. Brooks, Daniel J. Caldwell, Kyungho Choi, Silke Hickmann, Elizabeth Innes, Kim Ostapyk, Jane P. Staveley, Tim Verslycke, Gerald T. Ankley, Karen F. Beazley, Scott E. Belanger, Jason P. Berninger, Pedro Carriquiriborde, Anja Coors, Paul C. DeLeo, Scott D. Dyer, Jon F. Ericson, François Gagné, John P. Giesy, Todd Gouin, Lars Hallstrom, Maja V. Karlsson, D. G. Joakim Larsson, James M. Lazorchak, Frank Mastrocco, Alison McLaughlin, Mark E. McMaster, Roger D. Meyerhoff, Roberta Moore, Joanne L. Parrott, Jason R. Snape, Richard Murray-Smith, Mark R. Servos, Paul K. Sibley, Jürg Oliver Straub, Nora D. Szabo, Edward Topp, Gerald R. Tetreault, Vance L. Trudeau, Glen Van Der Kraak

**Affiliations:** 1 Environment Department, University of York, Heslington, York, United Kingdom; 2 Baylor University, Waco, Texas, USA; 3 Johnson and Johnson, New Brunswick, New Jersey, USA; 4 Seoul National University, Seoul, South Korea; 5 Umweltbundesamt, Dessau, Germany; 6 Health Canada, Ottawa, Ontario, Canada; 7 Exponent, Cary, North Carolina, USA; 8 Gradient, Cambridge, Massachusetts, USA; 9 U.S. Environmental Protection Agency, Duluth, Minnesota, USA; 10 Dalhousie University, Halifax, Nova Scotia, Canada; 11 Procter & Gamble, Cincinnati, Ohio, USA; 12 Centro de Investigaciones del Medio Ambiente, Facultad de Ciencias Exactas, Universidad Nacional de la Plata, La Plata, Argentina; 13 ECT Oekotoxikologie GmbH, Floersheim/Main, Germany; 14 American Cleaning Institute, Washington, DC, USA; 15 Pfizer Inc., New York, New York, USA; 16 Environment Canada, Montreal, Quebec, Canada; 17 University of Saskatchewan, Saskatoon, Saskatchewan, Canada; 18 Unilever, Colworth, Bedfordshire, United Kingdom; 19 University of Alberta, Edmonton, Alberta, Canada; 20 Sahlgrenska Academy at the University of Gothenburg, Gothenburg, Sweden; 21 U.S. EPA Office of Research and Development, Cincinnati, Ohio, USA; 22 Environment Canada, Burlington, Ontario, Canada; 23 Eli Lilly and Company, Indianapolis, Indiana, USA; 24 AstraZeneca UK Ltd., Brixham, Devon, United Kingdom; 25 University of Waterloo, Waterloo, Ontario, Canada; 26 University of Guelph, Guelph, Ontario, Canada; 27 F. Hoffmann-La Roche Ltd., Basel, Switzerland; 28 Department of Biology, University of Ottawa, Ottawa, Ontario, Canada; 29 Agriculture and Agri-Food Canada, London, Ontario, Canada

**Keywords:** antibiotic resistance, ecotoxicity, exposure assessment, health effects, personal care products, pharmaceuticals, prioritization, risk assessment, risk management

## Abstract

Background: Over the past 10–15 years, a substantial amount of work has been done by the scientific, regulatory, and business communities to elucidate the effects and risks of pharmaceuticals and personal care products (PPCPs) in the environment.

Objective: This review was undertaken to identify key outstanding issues regarding the effects of PPCPs on human and ecological health in order to ensure that future resources will be focused on the most important areas.

Data sources: To better understand and manage the risks of PPCPs in the environment, we used the “key question” approach to identify the principle issues that need to be addressed. Initially, questions were solicited from academic, government, and business communities around the world. A list of 101 questions was then discussed at an international expert workshop, and a top-20 list was developed. Following the workshop, workshop attendees ranked the 20 questions by importance.

Data synthesis: The top 20 priority questions fell into seven categories: *a*) prioritization of substances for assessment, *b*) pathways of exposure, *c*) bioavailability and uptake, *d*) effects characterization, *e*) risk and relative risk, *f* ) antibiotic resistance, and *g*) risk management.

Conclusions: A large body of information is now available on PPCPs in the environment. This exercise prioritized the most critical questions to aid in development of future research programs on the topic.

Pharmaceuticals and personal care products (PPCPs) include numerous chemical classes. Pharmaceuticals are used primarily to prevent or treat human and animal disease, whereas personal care products are used to improve the quality of daily life and include products such as moisturizers, lipsticks, shampoos, hair colors, deodorants, and toothpastes. Human-use PPCPs are generally excreted and emitted into the sewerage system following use. The compounds may then be released into surface waters or enter terrestrial systems when sewage effluent is used for irrigation or where sewage sludge is applied as a fertilizer to agricultural land (Kinney et al. 2006; Ternes et al. 2004). Veterinary pharmaceuticals are released to the environment either directly, from use in aquaculture and the treatment of pasture animals, or indirectly during the land application of manure and slurry from livestock facilities (Boxall et al. 2003a). PPCPs may also be released to the environment from manufacturing sites (Fick et al. 2009).

PPCPs have been detected in the natural environment across the world (e.g., Hirsch et. al. 1999; Kolpin et al. 2002; Ramirez et al. 2009). Although reported concentrations are generally low, many PPCPs have been detected in a variety of hydrological, climatic, and land-use settings and some can persist in the environment for months to years (e.g., Monteiro and Boxall 2009). Pharmaceuticals, as well as several chemicals used in personal care products, are biologically active compounds that are designed to interact with specific pathways and processes in target humans and animals. Concerns have therefore been raised about the potential effects of active PPCPs in the environment on human and environmental health; over the past 15 years, a substantial amount of work has been done to determine the occurrence, fate, effects, and risks of PPCPs in the environment. Regulations have also been developed regarding the assessment of risks of environmental exposure to PPCPs [e.g., Center for Drug Evaluation and Research (CDER) 1998; Committee for Medicinal Products for Human Use (CHMP) 2006; Committee for Medicinal Products for Veterinary Use (CVMP) 2000, 2004; European Centre for Ecotoxicology and Toxicology of Chemicals (ECETOC) 2008; World Health Organization (WHO) 2011].

Attempts have been made to synthesize the wealth of knowledge gained to date and to identify the remaining major research gaps and gaps in regulation [e.g., Knowledge and Need Assessment of Pharmaceutical Products in Environmental Waters (KNAPPE) 2008]. However, these exercises have tended to focus on select regions of the world, as well as established markets, and have not always engaged fully with major stakeholder groups.

One approach to identifying key issues in a topic area is to perform a “key question exercise,” which is designed to promote engagement of researchers and stakeholders from a broad range of sectors (e.g., Fleishman et al. 2011; Rudd et al. 2011). The exercise begins with an initial solicitation of interested parties to develop a list of questions that individual members of the community feel are important regarding a particular topic. A workshop is then held to discuss and prioritize the questions raised and to develop a final list of questions (e.g., 20, 40, or 100). In this review we report the results of a key question exercise that was performed to identify and rank the top 20 questions related to the hazards, exposure assessment, and environmental and health risks of PPCPs in the natural environment. A description of the approach used, the submitted questions, and questions taken forward to the workshop is available in Supplemental Material, pp. 2–29 (http://dx.doi.org/10.1289/ehp.1104477).

## Top 20 Questions

The top 20 questions fell into seven categories: *a*) prioritization of PPCPs, *b*) pathways of exposure, *c*) bioavailability and uptake, *d*) effects characterization, *e*) risk and relative risk, *f* ) antibiotic resistance, and *g*) risk management.

### Prioritization of PPCPs

*What approaches should be used to prioritize PPCPs for research on environmental and human health exposure and effects?* More than 4,000 pharmaceuticals are currently in use, and many types of chemicals are used in personal care products; it would be impossible to experimentally assess the hazards and risks of all of these in a timely manner. Prioritization approaches can be used to focus monitoring, testing, and research resources and to identify those PPCPs that are likely to pose the greatest risk in a particular situation. Several prioritization methods have been proposed for—and applied to—human pharmaceuticals (e.g., Kostich et al. 2010; Kostich and Lazorchak 2007; Sanderson et al. 2004) and veterinary medicines (Boxall et al. 2003b; Capelton et al. 2006; Kools et al. 2008). Many of these approaches use either exposure and toxicological predictions or information on pharmaceutical potency, so they can be readily applied to large numbers of compounds. These approaches should be further developed for different situations covering different geographical regions, climates, demographics, and cultural backgrounds and should be designed in such a way that they account for the use practices, complex fate processes, and the specific modes of action associated with many PPCPs.

### Pathways of Exposure

**Figure 1:**
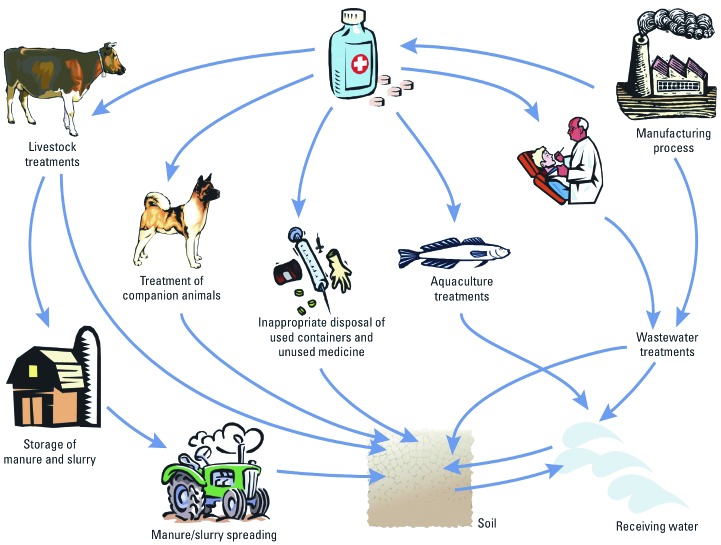
Major pathways of PPCP release into the environment. Reproduced from Boxall (2004) with permission from *EMBO* Reports.

*What are the environmental exposure pathways for organisms (including humans) to PPCPs in the environment, and are any of these overlooked in current risk assessment approaches?* PPCPs can enter the environment by a number of pathways (Figure 1). Regulatory environmental risk assessment approaches for PPCPs consider releases to surface waters from wastewater treatment systems, aquaculture facilities, and runoff from fields, as well as releases to soils during biosolid and manure application [e.g., CHMP 2006; CVMP 2008; Price et al. 2010). Other exposure pathways exist, including emissions from manufacturing sites (Fick et al. 2009), disposal of unused medicines to landfills, runoff of veterinary medicines from hard surfaces in farmyards, irrigation with wastewater, off-label emissions, and disposal of carcasses of treated animals. Management and use practices in different regions of the world can also vary, so an important exposure pathway in one geographical area may be a less important pathway in another region. For example, in several regions of the world, the connectivity of the population to wastewater treatment technologies is limited, so regulatory exposure modeling based on European and North American systems will not always be relevant. An understanding of the release mechanisms and dominant exposure pathways for PPCPs in different regions is therefore needed.

### Bioavailability and Uptake

*How can the uptake of ionizable PPCPs into aquatic and terrestrial organisms and through food chains be predicted?* A significant proportion of PPCPs are ionizable substances. Although methods are available for estimating uptake of ionizable compounds into fish and invertebrates (e.g., Fu et al. 2009; Meredith-Williams et al. 2012), our understanding of the factors and processes that influence uptake of PPCPs from different environmental compartments into organisms is still less well developed than for nonionizable chemicals (Brooks et al. 2009). The uptake of ionizable PPCPs is also very sensitive to changes in environmental conditions such as pH and soil and sediment characteristics. Data on uptake through food chains is almost nonexistent. Future work should therefore focus on understanding the uptake routes for PPCPs from a range of matrices into single organisms and food webs covering different traits (e.g., size, life cycle characteristics, method of respiration). Based on these studies, improved models should be developed for estimating uptake of ionizable PPCPs into organisms and through food chains.

*What is the bioavailability of nonextractable residues of PPCPs?* Many PPCPs dissipate rapidly in animal manure, biological treatment processes, soils, and sediments. Data from degradation studies with radiolabeled PPCPs indicate that, in many instances, the observed dissipation can be due to the formation of nonextractable residues (NERs; e.g., Kreuzig and Höltge 2005). NERs are species of a chemical that cannot be extracted from a matrix (sediment, soil, etc.) by methods that do not significantly change the chemical nature of the residues. In general, the chemical identities of these NERs are unknown. Concerns have been raised that, in the future, NERs may become bioavailable due to breakdown of manure and biosolid material added to soils, or due to changes in agricultural practices or the environment, such as changes in the pH or ionic strength of a system (Barraclough et al. 2005; Gevao et al. 2000). The challenge is to demonstrate whether NERs for PPCPs are bioavailable or whether they are likely to become bioavailable. This is a challenge not only for PPCP risk assessment but also for other classes of chemicals, including pesticides (e.g, Calderbank 1989; ECETOC 2010).

### Effects Characterization

*How can pharmaceutical preclinical and clinical information be used to assess the potential for adverse environmental impacts of pharmaceuticals?* A lot of information is available from mammalian studies and clinical trials on the behavior and effects of active pharmaceutical ingredients. The pharmaceutical industry devotes significant resources to collating new and emerging data as part of their postauthorization pharmacovigilance programs, and several epidemiological studies have been performed to explore the potential long-term health effects of pharmaceuticals on workers in the pharmaceutical industry (e.g., Heron and Pickering 2003). In contrast, comprehensive information on fate and effects in the environment is publicly available for only a small percentage of pharmaceuticals and, with a few exceptions (e.g., the U.K. Veterinary Medicines Directorate Suspected Adverse Reactions Reporting Scheme), pharmacovigilance programs do not examine environmental effects. By accessing the wealth of data from mammalian studies and clinical trials and by building upon the advanced methods for predicting long-term, low-level effects arising from occupational exposure, it may be possible to establish whether low levels of a pharmaceutical in the environment constitute a threat to environmental and human health (Ankley et al. 2007; Berninger and Brooks 2010; Huggett et al. 2003; Seiler 2002).

*What can be learned about the evolutionary conservation of PPCP targets across species and life stages in the context of potential adverse outcomes and effects?* Most pharmaceuticals, and a few personal care products, are designed to interact with a target (such as a specific receptor, enzyme, or biological process) in humans and animals to deliver the desired therapeutic effect. If these targets are present in organisms in the natural environment, exposure to some PPCPs might be able to elicit effects in those organisms. Knowledge of the presence or absence of PPCP targets across a wide range of taxa could therefore be invaluable in identifying PPCPs that might affect the environment at low concentrations, and those organisms and life stages of organisms that are most likely to respond to exposure to a particular pharmaceutical (Ankley et al. 2007; ECETOC 2008; Gunnarsson et al. 2008; Huggett et al. 2003; Seiler 2002; Trudeau et al. 2005). Comparative biochemistry, genomics, and other “omic” technologies offer potential tools for identifying PPCPs of potential concern, as well as the most sensitive and vulnerable species.

**Figure 2:**
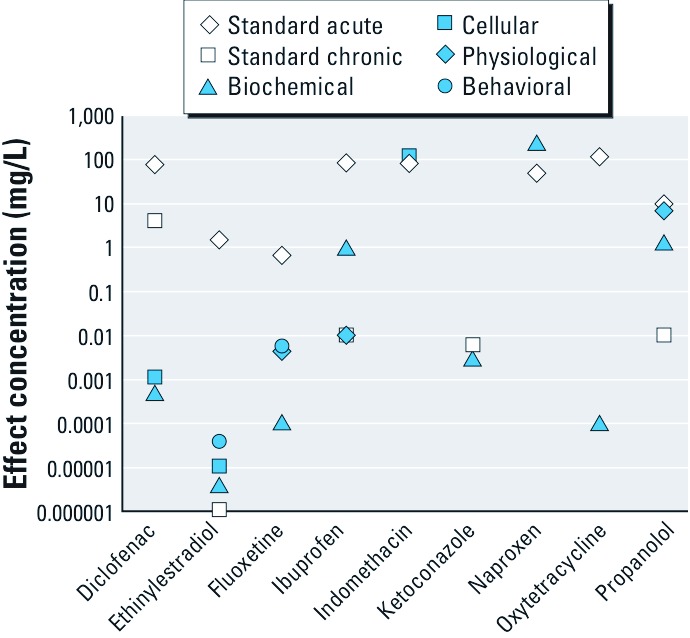
Relationship between results of acute and chronic studies (recommended for use in current regulatory assessment approaches for PPCPs in fish) and reported non­standard end points in fish and invertebrates. Standard acute and chronic data (e.g., fish and invertebrate mortality, reproduction, and growth) were obtained from FASS.se (2011) and several litera­ture sources (Caldwell et al. 2008; Kim et al. 2009; Yamamoto et al. 2007); non­standard end point data were obtained from Corcoran et al. (2010).

*How can ecotoxicological responses, such as histological and molecular-level responses observed for PPCPs, be translated into traditional ecologically important end points, such as survival, growth, and reproduction of a species?* This question is relevant to many other classes of environmental contaminants (Huggett et al. 1992). Responses such as histological changes, behavioral effects, biochemical responses, and up- or down-regulation of genes have been observed in organisms exposed to PPCPs (Ankley et al. 2007; Brooks et al. 2009; Corcoran et al. 2010). These responses are generally not included in current risk assessment schemes but can occur at concentrations that are orders of magnitude lower than concentrations at which effects are observed in regulatory tests, such as acute studies examining effects on fish and invertebrate mortality, or chronic studies looking at effects on reproduction and growth (Figure 2). The importance of these responses in terms of population survival and ecosystem functioning is poorly understood. However, it is necessary to understand these relationships in order to discover the broader implications of nonstandard observations on ecosystem health and to determine the benefits of incorporating data from nonstandard ecotoxicological responses, such as histological and behavioral effects, into prospective and retrospective risk assessment frameworks. Unlike for many other chemical classes, our knowledge of the relationships between effects at the molecular level and effects at the whole-organism level in humans is very well developed. We may therefore be able to apply this knowledge to better understand relationships between molecular, cellular, and whole-organism end points for organisms in the natural environment.

*How can ecotoxicity test methods that reflect the different modes of action of active PPCPs be developed and implemented in customized risk assessment strategies?* Existing risk assessment approaches for PPCPs in Europe and North America employ standard Organisation for Economic Co-operation and Development (OECD) test methods for examining effects on organisms (CDER 1998; CHMP 2006; CVMP 2000, 2004). Some authorities can ask for nonstandardized studies when a risk cannot be ruled out by standard tests. Concerns have been raised over whether standard methods will identify ecologically important effects of specifically acting PPCPs (Brooks et al. 2009; ECETOC 2008). The effect of the nonsteroidal, anti-inflammatory compound diclofenac on vulture populations (Oaks et al. 2004) is one example of an end point that would not have been predicted from standard studies. Further work is required to understand the effects of PPCPs with different modes of action on aquatic and terrestrial organisms. Depending on the findings of this research, it may be appropriate to develop new guidance on the selection of ecotoxicological test methods (species and end points) in the risk assessment process. However, it would be shortsighted to restrict testing strategies only to methods that reflect specific modes of action because unexpected effects in organisms can occur, as illustrated by the high potency of fluoxetine, a selective serotonin reuptake inhibitor, in algae (Oakes et al. 2010).

*How can effects from long-term exposure to low concentrations of PPCP mixtures on nontarget organisms be assessed?* Aquatic and terrestrial systems will be exposed to a complex mixture of PPCPs and other contaminants. Many pharmaceuticals, if consumed together at therapeutic doses, can cause severe adverse interactions in humans (e.g., Juurlink et al. 2003). If aquatic organisms respond to these compounds in the same way as humans, effects on the environment could be greater than predicted based on effects data for the single compounds. Antimicrobial PPCPs may also increase persistence of other PPCPs, thus affecting the overall risk (Monteiro and Boxall 2009). Because many human-use PPCPs will be emitted continuously into the environment, organisms in the environment will be exposed throughout their lifetime. However, no regulatory program for prospective environmental risk assessment of PPCPs (or other product classes) takes into account the long-term combined toxicity of mixtures of chemicals, so there is a need to develop new approaches for assessing the risks arising from long-term exposure to mixtures. The concept of mixture risk assessment is gathering momentum, particularly in the public health arena, and recent reports by the European Commission, the UK Committee on Toxicology, and the U.S. National Academy of Sciences have already started to consider this topic (e.g., Kortenkamp et al. 2009). For human medicines, it may be possible to use observed contraindications in humans to provide an indication of whether a particular combination of pharmaceuticals in the environment may be of concern. Mixture interactions could also be simulated by pharmacokinetic modeling, linking models at the interaction site (Krishnan et al. 2002), although this will require extensive quantitative information on pharmacokinetics and/or toxicokinetics. Although the use of *in vitro* assays for relevant end points (e.g., carcinogenic, mutagenic, and reproductive effects) to assess the effects of mixtures of pharmaceuticals that typically occur in environmental systems may also provide useful information for use in risk assessment, these will need to be extensively validated before use.

*Can nonanimal testing methods be developed that will provide equivalent or better hazard data compared with current* in vivo *methods?* For personal care products, there is regulatory pressure in some geographic regions to reduce the amount of animal testing used for human safety and environmental risk assessment in a 3Rs framework (reduce, refine, replace). It may be possible to reduce the amount of animal testing using nonanimal testing methods, such as *in vitro* approaches and *in silico* methods (e.g., quantitative structure–activity relationships, read-across and expert systems), by optimizing experimental designs, and by employing intelligent testing strategies (Hutchinson et al. 2003; OECD 2010; Rufli and Springer 2011). Although these approaches are being promoted (e.g., National Academy of Sciences 2007) and used for industrial chemicals [e.g., as part of the REACH (Registration, Evaluation, Authorisation and Restriction of Chemical substances) regulations in Europe and elsewhere (Halder et al. 2010)], additional approaches are needed to replace animal test methods with methods able to evaluate specific and nonspecific modes of action.

### Risk and Relative Risks

*How can regions where PPCPs pose the greatest risk to environmental and human health, either now or in the future, be identified?* Risks of PPCPs in the environment in different geographic regions vary because of differences in the presence/absence and type of manufacturing sites, level of PPCP use, population demographics, cultural practices, environmental and climatic characteristics, dilution potential of receiving environments, and infrastructure related to wastewater and drinking water treatment. Risks may change in the long term due to factors such as increased urbanization and effluent-dominated instream flows (Brooks et al. 2006), increased disease pressures, demographic change, population increases, technological developments (e.g., move from small molecules to biologics, development of nanomedicines, improvements in drug delivery), and climate change. By better understanding the drivers for PPCP exposure in different regions, it may be possible to identify those areas that are at greatest risk, meaning that control options can be focused to areas/regions where they will be most effective. By understanding how risks will change in the longer term, it may be possible to anticipate and preemptively mitigate against unacceptable changes in risks.

*How important are PPCPs relative to other chemicals and nonchemical stressors in terms of biological impacts in the natural environment?* PPCPs are released into the natural environment along with many other chemicals (e.g., nutrients, metals, industrial chemicals, pesticides, natural hormones). The natural environment is also exposed to nonchemical stressors such as changes in water flow and temperature. The affect of PPCPs could be small compared with the many other chemical and nonchemical stressors present in the natural environment. To make informed management decisions, it is necessary to understand the relative impact of PPCPs compared with other pressures in a particular situation.

*Do PPCPs pose a risk to wildlife such as mammals, birds, reptiles, and amphibians?* Most studies have focused on effects of PPCPs on fish and invertebrates, but our knowledge of risks to other wildlife species, such as birds and small mammals, is less developed. Several case studies have highlighted the importance of understanding effects on birds and mammals. For example, the inappropriate use of diclofenac and associated cultural practices regarding disposal of animal carcasses, combined with the high sensitivity of vultures to diclofenac, were responsible for the decline in populations of three vulture species in Asia (Oaks et al. 2004), resulting in ecological, socioeconomic, cultural, and human health impacts (Markandya et al. 2008). Indirect effects on top predators may also be important; for example, there is concern that antiparasitic veterinary medicines may be indirectly affecting populations of insect-eating bats and birds by affecting the quantity of food available (McCracken 1993). More work is needed to better understand the exposure of birds, mammals, and amphibians to PPCPs, as well as the potential toxicological effects of PPCPs on these species.

*How can the environmental risks of metabolites and environmental transformation products of PPCPs be assessed?* Pharmaceuticals may be metabolized in the treated human or animal so that a mixture of parent compound and metabolites will be released into the environment. Transformation of PPCPs will also occur in wastewater treatment processes, surface waters, sediments, manure, soils, and drinking water treatment processes (Escher and Fenner 2011). Although metabolites and transformation products are usually less hazardous than the parent compound, data for pesticides indicate that some can be more toxic (Sinclair and Boxall 2003). The environmental fate of these substances can also be different from the parent compound, meaning that environmental compartments that are not exposed to the parent may be exposed to a transformation product (Boxall et al. 2004). Concerns have also been raised over the potential human health effects of selected transformation products of PPCPs, such as the halogenated and nitrosamine products resulting from transformation in wastewater and drinking water treatment processes (Sedlak and von Gunten 2011). We need to better understand the release and formation of transformation products of PPCPs in the environment and develop approaches for identifying transformation products that could pose a greater risk than the parent compound.

*How can data on the occurrence of PPCPs in the environment and quality of ecosystems exposed to PPCPs be used to determine whether current regulatory risk assessment schemes are effective?* Environmental risk assessments for PPCPs have been required in Europe and North America for some time. The effectiveness of these prospective risk assessment approaches, in terms of predicting exposure and effects in the real world, is not always clear. By bringing together data on the occurrence of PPCPs in different regions, as well as information on the status of biological communities and ecosystems, it may be possible to establish whether environmental risk assessment schemes really work for PPCPs. This is a general issue relevant to other classes of chemicals that require an environmental risk assessment. The application of ecoepidemiological approaches that link chemical pressures to effects on ecosystems (e.g., De Zwaart et al. 2006) may help answer this question.

### Antibiotic Resistance

*Does environmental exposure to PPCP residues result in the selection of antimicrobial-resistant microorganisms, and is this important in terms of human health outcomes?* The WHO (1998) identified the emergence of antimicrobial resistance as one of the serious concerns of health policies in the future. One of the major concerns relating to the occurrence of antibiotic compounds in the environment is the potential for selection of resistant microbial species. Antibiotics in the environment may enhance the formation of single, cross-, and multiple-resistance in bacteria (e.g., Byrne-Bailey et al. 2009; Gaze et al. 2011; Knapp et al. 2010; Kristiansson et al. 2011). However, the role of environmental residues of antibiotics in the selection of antibiotic resistance is still unclear; even where information exists, it is only available for a few antibiotics (e.g., sulfonamides, fluoroquinolones). We need to better understand how antibiotic residues in the environment are involved in selecting for antibiotic resistance, as well as the potential for the acquired resistance to transfer to human and animal pathogens and thus affect human health. This assessment must be interpreted against the backdrop of antibiotic resistance (the resistome) naturally found in the environment or caused by inappropriate clinical use of antibiotics or other environmental contaminants.

*How can the risks to human health that arise from antibiotic resistance selection by PPCPs in the natural environment be assessed?* Current regulatory paradigms do not consider the potential for antibiotic resistance selection in soils and surface waters. If the occurrence of antibiotics in the natural environment is demonstrated to be an important driver for resistance selection, it may be necessary to develop approaches for considering resistance selection in the natural environment as an end point in the safety assessment of new antibiotic substances. There is also a need to understand the extent to which feces from treated animals and humans act as an environmental source of resistant microorganisms and associated genetic elements.

### Risk Management

*If a PPCP has an adverse environmental risk profile, what can be done to manage and mitigate the risks?* In the event that a PPCP poses an unacceptable risk to the environment, options exist for minimizing or removing emissions to the environment, including *a*) substitution of the compound with a more environmentally benign compound; *b*) development of better drug delivery systems so that smaller doses are needed; *c*) improvement of packaging and package sizes to extend shelf life and reduce the amount of the product that expires and must be discarded unused; *d*) changes in prescription and animal husbandry practices; and *e*) introduction of improved wastewater treatment options (e.g., Daughton 2003a, 2003b; START 2008). However, the efficacy and practicality of many of these solutions is poorly understood. A systematic study is needed to determine the benefits of different management and mitigation options and any societal and environmental costs associated with a particular option in different regions of the world. This will allow informed decisions to be made on the best mitigation strategy.

*What effluent treatment methods are effective in reducing the effects of PPCPs in the environment while not increasing the toxicity of whole effluents?* During biological treatment, some PPCPs may be degraded or removed through sorption to sludge (Prasse et al. 2011; Ternes et al. 2004). Recalcitrant PPCPs may be removed using tertiary treatment methods such as ozonation, activated carbon adsorption, or nanofiltration (Ternes et al. 2004). In some cases, the wastewater treatment process may increase the risk. For example, ozonation may result in the formation of more toxic oxidation products. In other cases, the introduction of a treatment option may move the exposure from one environmental compartment to another. For example, introduction of procedures to enhance sorption of PPCPs to activated sludge treatment will mean that while emissions to water bodies are reduced, exposure of the terrestrial environment will increase when the sludge is applied to soils as a fertilizer (McClellan and Halden 2010). Increased knowledge is required to determine the effectiveness and consequences of waste and drinking water treatment options on PPCP fate and effects.

*How can the efficacy of risk management approaches be assessed?* The introduction of risk management strategies can result in environmental, economic, and societal costs. In these cases, management options should be proven to be effective at reducing environmental impacts before they are widely introduced. Guidance is needed in evaluating environmental monitoring approaches to determine the efficacy of particular management options. These approaches should not only include monitoring of changes in the occurrence of a particular substance but also be able to monitor changes (improvements) in the health of the ecosystem of interest. The costs (economic, social, and environmental) of a management option also need to be considered. Control options for antimicrobial compounds (e.g., banning of antibiotic substances as growth promoters and/or prophylactic treatments in agriculture) may not be effective in controlling antibiotic resistance because once antibiotic resistance genes are present in the environment, they may not disappear. Other anthropogenic compounds will also facilitate the selection of microrganisms that are resistant to some classes of antibiotic (e.g., Gaze et al. 2011).

## Ranking of Questions and Next Steps

When questions were ranked in terms of importance, the question regarding the relative risks of PPCPs compared with other environmental stressors was identified as the most important (Table 1). This reflects the significance of the question for allocation of future research resources and the implementation of future policy development and risk management options. If the answer to the question is that PPCPs are more important stressors than are other stressors in the environment, then many other questions identified in this exercise will be relevant and important. However, if PPCPs pose relatively minor risks compared with other stressors in the environment, then expending large amounts of resources answering the other questions may not offer the best use of resources in terms of global environmental protection. Questions regarding prioritization, improved characterization of effects, and antibiotic resistance were also ranked highly, whereas questions regarding risks of nonextractable residues, treatment, and the use of nonanimal studies were ranked lower (Table 1). For each question, potential approaches that could be adopted to address the question were identified. We envisage that this information will be invaluable in formulating future research programs involving the risks of PPCPs in the environment.

**Table 1:** Ranking of key questions by workshop participants, along with potential approaches to address the questions, the interrelationships of the questions, and the degree of challenge required to address a question.

Rank	Question	Most important question (%)a	Potential approaches	Related questions (by rank)	Level of challenge
1	How important are PPCPs relative to other chemicals and nonchemical stressors in terms of biological impacts in the natural environment?		48.5		Comparative assessment of risks posed by PPCPs compared with other stressors; effects-driven analysis on ecologically important end points for effluent samples to identify the relative toxicity of the chemical components; ecoepidemiological studies		Will inform whether resources should be expended on many other questions, particularly those around risk management (e.g., 14); data from 2, 3, 6, and 17 may help to answer the question		M–H
2	What approaches should be used to prioritize PPCPs for research on environmental and human health exposure and effects?		35.8		Review of existing prioritization approaches to identify advantages and limitations and geographical representativeness; development and application of new approaches for different scenarios and regions; review of existing prioritization approaches to determine whether similar PPCPs are highlighted against different prioritization metrics		5, 6, 9, and 19 may provide useful data		L–M
3	Does environmental exposure to PPCP residues result in the selection of antimicrobial resistant microorganisms, and is this important in terms of human health outcomes?		34.3		Large-scale multidisciplinary studies to characterize the impacts of antibiotic residues on resistance in treatment systems, surface waters and soils; characterization of the degree of human exposure to resistance genes arising from the natural environment; comparison of antibiotics in the environment with other pressures, such as selection in the clinical setting and selection by other contaminants		Could help inform 11		H
4	How can ecotoxicological responses, such as histological and molecular-level responses observed for PPCPs, be translated into traditional ecologically important end points such as survival, growth, and reproduction of a species?		32.7		Generation of data on effects of a range of PPCPs on organisms at different levels (biomarker through populations); use of organism and population models to attempt to explain the linkages		2 could inform which substances to focus on; information from 6 may help to answer this question		H
5	How can pharmaceutical preclinical and clinical information be used to assess the potential for adverse environmental impacts of pharmaceuticals?		32.4		Development of comparative datasets on preclinical, clinical, and ecotoxicological data for a range of substances with different modes of action and physicochemical properties; evaluation of datasets to pull out major relationships		Information from 6 may explain different responses of humans and ecosystems		M
6	What can be learned about the evolutionary conservation of PPCP targets across species and life stages in the context of potential adverse outcomes and effects?		31.1		Increased knowledge about the conservation of drug targets across environmental phyla and taxa through increased genome coverage; application of an adverse outcome pathway approach to understand relationships between target interactions and adverse effects on ecosystems		4		H
7	How can effects from long-term exposure to low concentrations of PPCP mixtures on nontarget organisms be assessed?		30.1		Large-scale ecoepidemiological studies; development of effective ecopharmacovigilance schemes; long-term well-controlled effects studies		5		H
8	How can ecotoxicity test methods that reflect the different modes of action of active PPCPs be developed and implemented in customized risk assessment strategies?		29.8		Development of strategies that integrate information on pharmacology, target conservation, and adverse outcome pathways to identify the best strategy for assessing the ecotoxicological effects of PPCPs		Could be informed by 4, 5, and 6.		L if other questions addressed
9	What are the environmental exposure pathways for organisms (including humans) to PPCPs in the environment, and are any of these overlooked in current risk assessment approaches?		25.7		Review of potential pathways of release of PPCPs to the environment at different stages of the product lifecycle for different regions of the world; analysis of existing risk assessment frameworks against this information; refinement of frameworks to include ignored exposure pathways where appropriate		Could help to inform 2 and 17.		L–M
10	How can the efficacy of risk management approaches be assessed?		23.8		Development of monitoring strategies (regarding use, disposal, occurrence, and impacts) at different stages of the product life cycle; some socioeconomic and cost-benefit analysis aspects should be included		None		M
Table continued									
Table 1. Continued.									
Rank	Question		Most important question (%)a		Potential approaches		Related questions (by rank)		Level of challenge
11	How can the risks to human health that arise from antibiotic resistance selection by PPCPs in the natural environment be assessed?		23.6		Development of risk assessment strategies; development of effective ecopharmacovigilance for antibiotics to assess the development and frequency of antibiotic resistance in natural microbial communities and clinical isolates		Information from 3 could be helpful in the development of risk assessment schemes		H
12	How can the uptake of ionizable PPCPs into aquatic and terrestrial organisms and through food chains be predicted?		22.4		Studies into the uptake, depuration, and metabolism of a range of ionizable PPCPs with different properties into water-, soil-, and sediment-dwelling organisms with different traits; food chain studies with selected substances; development of uptake models		May help inform 1 and 15		L–M
13	How can data on the occurrence of PPCPs in the environment and quality of ecosystems exposed to PPCPs be used to determine whether current regulatory risk assessment schemes are effective?		22.1		Collation of data on the occurrence of PPCPs in receiving systems and on associated ecology; analysis of data against exposure predictions from environmental risk assessments; evaluation of quality of ecosystems receiving PPCPs; when impacts cannot be ruled out, it will be necessary to tease out the impacts of PPCPs on a system against impacts of other stressors.		None		M–H
14	If a PPCP has an adverse environmental risk profile, what can be done to manage and mitigate the risks?		19.0		Review of different management and mitigation options for different stages of the product life cycle; generation of data on the efficacy of a particular option; assessment of economic and other implications of an option so that benefits of a system can be weighed up against the potential costs		Could be informed by data from 1, 3, and 10.		M
15	Do PPCPs pose a risk to wildlife such as mammals, birds, reptiles and amphibians?		17.1		Development of exposure models for birds, amphibians, and mammals; evaluation of toxic effects of PPCPs on birds, mammals, and amphibians using either existing preclinical data or well designed studies; use of environmental monitoring studies		Data from 5, 9, and 12 may be useful		M–H
16	How can the environmental risks of metabolites and environmental transformation products of PPCPs be measured or predicted?		17.1		Development of improved analytical approaches for identifying metabolites and transformation products; studies to assess relative effects of transformation products compared with parent compounds; development of assessment schemes for transformation products		Information from 5 may help		L–M
17	How can regions where PPCPs pose the greatest risk to environmental and human health, either now or in the future, be identified?		16.2		Evaluation of usage patterns of PPCPs in different geographical regions, as well as local practices (e.g., for disposal and treatment of contaminated material) and potential differences in sensitivity of organisms, both for now and in the future; development of new exposure assessment models if appropriate; use of information to establish potential risks		Data from 9 will be useful		L–M
18	What effluent treatment methods are effective in reducing the effects of PPCPs in the environment while not increasing the toxicity of whole effluents?		16.2		Targeted laboratory and field studies, which consider local conditions and constraints, to determine how PPCPs are removed in treatment processes and whether transformation products are formed; use of biologically based assessments to assess effectiveness of a particular treatment method		Information from 4 and 8 could assist in the selection of biological end points to use; data from 16 may help		M
19	Can nonanimal testing methods be developed that will provide equivalent or better hazard data compared with current in vivo methods?		13.2		Review of current nonanimal methods; assessment of information from selected methods against data from current in vivo methods; development of recommendations on which nonanimal methods can provide useful data		Knowledge from 5 may help		M
20	What is the bioavailability of nonextractable residues of PPCPs?		9.4		Improved analytical characterization of the form of PPCP NERs; controlled studies on the bioavailability of NERs of a range of PPCPs to soil- and sediment-dwelling organisms with different traits over time; manipulation studies to assess the impacts of climate change, for example, on the availability of NERs; development of guidelines for NER assessment in risk assessment		None		L
Abbreviations: H, high (likely to require large, complex, multidisciplinary research programs and development of new paradigms); M, medium (likely to require large, multidisciplinary programs but many of the required tools exist; L, low (readily addressable through focused research programs). aWorkshop attendees were sequentially presented with sets of 4 questions and asked to select the highest (most important) and lowest (least important) question from the each set; all 20 questions were ranked using this process. Values correspond to the proportion of instances that a question was ranked by the attendees as highest in the set of four questions.									

In the development of future research and policy initiatives, it is important to recognize that many of the questions are interrelated and that knowledge gained from one question may be needed to address other questions (Table 1). In some instances, it may be necessary to address a lower-ranked question before a high-ranked question can be fully answered. For example, knowledge gained from answering questions around prioritization of PPCPs, the importance of the environment as a selection pressure for antimicrobial resistance, identification of regions of greatest risk, and characterization of risks to wildlife may all need to be answered before the top-ranked question can be addressed. The level of challenge associated with answering a question also varies (Table 1). Questions regarding the risks of nonextractable residues and prioritization of PPCPs may be addressable with limited investment over relatively short time periods compared with other questions.

Many questions (e.g., the questions involving NERs, mixture interactions, and extrapolation of results on molecular and cellular effects to ecologically relevant end points) are not unique to PPCPs, so it may be appropriate to address these in broader research programs looking at other chemical classes. However, the detailed understanding that we have for the effects of many PPCPs on humans might make them good candidate model compounds for addressing some of these wider questions.

## Wider Global Relevance of the Exercise

Although participants from different regions of the world were engaged in the exercise, most question submissions (92%) were from North America (46.6%), Europe (29.8%), and Australia/New Zealand (15.5%). The workshop was also dominated by North American (51%) and European attendees (14.6%) and representatives of multinational corporations (34%). Two subsequent regional workshops were held in South Korea and Australia to determine the relevance of the 20 questions to the East Asian and Australia/New Zealand regions, respectively, and to identify additional questions that may be important to these regions. Participants at these workshops agreed that the 20 questions were of high importance to the East Asian and Australasian regions, and they highlighted the fact that these regions had unique characteristics (e.g., in terms of biodiversity) that should be considered when addressing the questions. Participants felt that important issues, such as better risk communication, consideration of cultural differences, and the impacts of natural medicines had also been overlooked. The conclusions of these later workshops will be presented in detail elsewhere.

## Conclusions

The present study is the first to use the key question approach to identify key issues regarding exposure, effects, and risks of PPCPs in the environment. We see this exercise as the start of a broader program, and in the short term we are planning a broader global survey to identify which questions are most relevant to different stakeholders and why (additional questions proposed by the “local” workshops will be included in this exercise). In addition to this survey, additional workshops are planned on select topics (e.g., antibiotic resistance), and the conclusions of the survey and the workshops will be disseminated to policy makers around the world. We are optimistic that the results of this exercise will be invaluable in informing the design, coordination, and implementation of future research programs on PPCPs in the environment. We hope that these programs will help us to better understand the potential and relative risks of these substances in the natural environment and to effectively control and manage these risks.

## Supplemental Material

(492 KB) PDF
