# Supplementary material for: Pharmaceuticals and Personal Care Products in the Environment: What Are the Big Questions?
Source: Environ Health Perspect. 2012 May 30;120(9):1221–9. doi: 10.1289/ehp.1104477 (PMC3440110; doi:10.1289/ehp.1104477)
Supplement: (492 KB) PDF [file ehp.1104477.s001.pdf]

Supplemental Material

Pharmaceuticals and Personal Care Products in the Environment: What are the Big Questions?

Alistair B.A. Boxall, Murray A. Rudd, Bryan W. Brooks, Daniel J. Caldwell, Kyungho Choi, Silke Hickmann, Elizabeth Innes, Kim Ostapyk, Jane P. Staveley, Tim Verslycke, Gerald T. Ankley, Karen F. Beazley, Scott E. Belanger, Jason P. Berninger, Pedro Carriquiriborde, Anja Coors, Paul C. DeLeo, Scott D. Dyer, Jon F. Ericson, François Gagné, John P. Giesy, Todd Gouin, Lars Hallstrom, Maja V. Karlsson, D. G. Joakim Larsson, James M. Lazorchak, Frank Mastrocco, Alison McLaughlin, Mark E. McMaster, Roger D. Meyerhoff, Roberta Moore, Joanne L. Parrott, Jason R. Snape, Richard Murray-Smith, Mark R. Servos, Paul K. Sibley, Jürg Oliver Straub, Nora D. Szabo, Edward Topp, Gerald R. Tetreault, Vance L. Trudeau, Glen Van Der Kraak

Contents

1. General Approach 2
2. Full question list submitted to the exercise 3
3. Questions taken forward to the workshop (broken down by breakout group) 24
4. References 30

1. General Approach

Our study followed the question solicitation and prioritization methodology outlined by Sutherland et al. (2011). Initially, invitations to submit candidate research questions to a dedicated question solicitation website were widely distributed. Individuals were told that an ideal question should easily be translated into a directly testable research hypothesis. The answers to the question should be relevant to decision-makers, in so far as they would increase the effectiveness of business and policy decisions about the development or management of PPCPs and their interactions with the environment. Six criteria were suggested for candidate questions, they should: 1) address important gaps in knowledge; 2) be answerable through a realistic research design; 3) have a factual answer that does not depend on value judgments; 4) cover a spatial and temporal scale that realistically could be addressed by a research team; 5) not be answerable simply by “it all depends” or a simple “yes” or “no”; and, 6) if related to impact and interventions, they should contain a subject, an intervention, and a measurable outcome.

A total of 403 candidate questions were received in 161 submissions from individuals working for private sector manufacturers (17), private sector consultants (13), government scientists (42), government regulators or policy makers (19), academics (61), non-governmental organization representatives (3), and individuals from other organizations (6). Geographically, 3 submissions were from Africa, 9 from Asia, 25 from Australia and New Zealand, 48 from 15 European countries, 1 from Latin America, and 75 from North America. Removal of questions that did not meet the aspirational criteria, which were outside the scope of the exercise, or which were replicated, left 101 questions.

The 101 questions were then discussed at a two-day workshop involving experts from a range of disciplines (including ecology, environmental chemistry, ecotoxicology, pharmacology and chemical risk assessment) with an interest in the risks of PPCPs in the environment. Participants represented government (11), business (13), and academia (17), with representation from individuals based in North America (20), Latin America (1), Europe (6), and Asia (1), and from companies or industry associations with global operations (13). Four of the 41 individuals did not have PPCP subject specialist knowledge but had participated in previous key question exercises and acted as recorders and facilitators for this workshop. Participants selected the top 20 questions using the process employed at North American top 40 exercises for biodiversity science research questions (Fleishman et al. 2011; Rudd et al. 2011). The workshop comprised plenary and breakout sessions. Candidate questions were grouped in loose themes, and individuals were assigned to breakout groups based on their technical specializations.

Each breakout group was assigned 8-15 candidate questions and tasked with culling the set to two top 20 recommendations and one alternate. In prioritising the questions, groups were asked to consider the aspirational criteria described above. At this stage, an additional aspirational criterion was introduced: i.e. questions should be addressable by a research project or programme within a finite budget and timeframe (e.g. $10 million over five years). Participants in breakout groups sometimes combined candidate questions and developed alternate questions that captured key ideas from candidate questions. The 26 questions that resulted from the first day were discussed during a facilitated plenary session on the second day, during which the top 20 were selected. A web-based survey of the participants was performed following the workshop in order to rank the 20 questions in terms of importance. Potential approaches to address the questions were also identified.

2. Full question list submitted to the exercise

1 Are cladoceran reproduction assays appropriate for characterizing chronic toxicity of all PPCPs?

2 Are herbivorous, omnivorous and piscivorous birds at risk from PPCP exposures?

3 Are PCPs and pharmaceuticals present at concentrations in various environmental compartments that can cause significant adverse effects on biota (i.e., what is the risk and how do they interact with other contaminants)?

4 Are plants or earthworms able to take up the pharmaceutical and personal care products in the terrestrial environments?

5 Are proposed approaches to leverage mammalian or target organism pharmacology and toxicology data to predict aquatic and terrestrial wildlife effects of PPCPs encouraging?

6 Are side effects of medicines appropriately assessed using current regulatory testing regimes?

7 Are the levels sufficient to cause an effect in aquatic or terrestrial environments? What are in the implications of the mixtures of pharmaceutical and personal care products in the environment?

8 Are the presence of pharmacological targets and their functions understood in aquatic plants, algae, invertebrates and vertebrates?

9 Are veterinary antibiotics and pharmaceuticals present in animal manure persistent and bio-available in the terrestrial environment and do they have the potential to move off-site?

10 Can guidance be given on how to assess the environmental risks associated with chemicals referred to as pseudo-persistent?

11 Can we use our special knowledge about the mechanisms of action of pharmaceuticals in mammals to understand any possible interactive effects of mixtures in fish?

12 Clarification with respect to the differences between chemicals used in personal care products and active ingredients used in pharmaceuticals, and implications regarding how an ERA is performed for the respective class of chemical compounds.

13 Compare the relative environmental profiles of Pharmaceutical and Personal Care products

14 Do ADME characteristics in mammals predict ADME of pharmaceuticals in fish models? For example, are Vd, Cmax, t1/2, Cl values from humans predictive of those parameters in a 700 g fish or a red-eared slider turtle?

15 Do low levels of active ingredients present any harm to the Biota?

16 Do pharmaceutical and personal care products persist in the biosolids, majority of which is disposed on land in Australia? What is the fate of biosolid bounds pharmaceutical and personal care products?

17 Do PPCP in the environment affect reproduction or development in exposed wildlife?

18 Do the low-level mixtures of pharmaceuticals present in surface waters and sediments pose a risk to aquatic species

19 Does environmental exposure to antimicrobial residues, or to antimicrobial-resistance determinants selected for anthropogenically, promote resistance in environmental microorganisms, and create a reservoir that is then important in resistance development in human pathogens or commensals?

20 Does more need to be done to educate the public with respect to the disposal of un-used medicines?

21 Does resistance pass from the natural environment to the clinical environment and result in hospital acquired infections?

22 Effects of statins in the aquatic environment, are there any chronic effects to non-target organisms? What are the mechanisms of toxicity? Differences in species sensitivity?

23 How can sewage treatment be improved to remove pharmaceutical loads into the environment?

24 How can drugs be designed so that they have shorter half-lives in the environment?

25 How can risk-benefit modelling be used as a tool to direct research and policy decisions.

26 How can the BCF and BAF of ionisable organics be measured or modelled?

27 How can the man on the street help to reduce pharmaceutical impacts on the environment?

28 How can the universe of "unknown unknowns" be best reduced?

29 How can we effectively target which areas or regions are most at risk to health issues related to pharmaceuticals in the environment? Are these risks related to manufacturing, use, population demographics or the presence or absence of infrastructure?

30 How can we make sure, that PPCPs do not enter the environment in concentrations that are able to cause adverse effects on the ecosystems?

31 How can we reduce emission of pharmaceutical waste in developing countries?

32 How can we validate the assumptions made in environmental risk assessment for the partitioning, uptake and metabolism of pharmaceuticals where Kow assumptions are not valid?

33 How could you test the effects of long-term low level exposure of humans to pharmaceuticals in the environment?

34 How do risks from trace levels of pharmaceutical and personal care products in the environment compare to other human and environmental risks facing society, and how can we quantify these risks for comparison.

35 How important is the contribution of pharmaceuticals compared to other contaminants to real-world mixture toxicity?

36 How should we manage the risks associated with pharmaceuticals that have environmental data gaps?

37 How will environmental change affect the use and fate of pharmaceuticals and personal care products and how will this affect environmental risks compared to today?

38 If a drug has an adverse PBT and/ or environmental risk profile what can be done to manage and mitigate the risks? Case studies?

39 If human health is at risk, how can we integrate human safety assessment and environmental risk assessment?

40 If the proposed approaches to leverage mammalian or target organism pharmacology and toxicology data to predict aquatic and terrestrial wildlife effects of PPCPs are promising, what data are needed to experimentally validate these potentially predictive models?

41 Improved methods and designing experiments to investigate ecotoxicological and risk assessment studies based on mixtures of organic contaminants is required. Can future research put more emphasis on this?

42 Is green drug design feasible? How will it work? And, what are the success criteria or protection goals? What if greener drug design is not feasible?

43 Is it appropriate to use Kow for ionisable organics, or would Dow be more appropriate as a trigger value within a regulatory context?

44 Is it possible to define the most sensitive species for testing of chemicals with specific modes of action?

45 Is the terrestrial compartment sufficiently protected by current ERA procedures?

46 Is there really a risk to human health through indirect exposure to pharmaceuticals?

47 Jurisdiction: How is it ensured that unused pharmaceuticals do not end up in the normal household waste? If they end up there what percentage will up end there?

48 Possibility of synergistic interaction of toxicity in mixture with contaminants other than pharmaceuticals.

49 Potential synergistic interaction in toxicity in mixture with other compounds including pharmaceuticals and chemicals other than pharmaceuticals

50 Should medicines with both human and veterinary applications be assessed within a single framework to capture the 'collective' risk?

51 Should regulatory assessments account for the influences of site specific pH on bioavailability and effects of weak acid and weak base PPCPs?

52 Should regulatory assessments of PPCPs account for differences in metabolism and effects of enantiomers? E.g., Why is chirality not accounted for in PPCP ERAs?

53 Should toxicity equivalency approaches / mixture models be developed to prospectively assess the effects of PPCPs with common MOAs that will co-occur in effluent discharges? e.g., would a "risk cup" approach be appropriate?

54 Should we collect the same amount of human health and environmental data on personal care products, and especially "natural" products, as we do for pharmaceuticals?

55 Should Whole Effluent Toxicity test methods be expanded to include fish reproduction, biomarkers, or alternative endpoints such as behaviour?

56 To what extent the sewage treatment plants are effective in removing these compounds. Is the treatment process leading to real solutions or shifting the problem from aquatic to terrestrial environment?

57 To what extent will the emission of antibiotics to the environment, by drug use or production, provoke the development of resistance?

58 What are appropriate risk assessment processes for whole effluent and mixtures? Should the chemical-by-chemical assessment approach be abandon or does it still have an appropriate place in an assessment paradigm?

59 what are endocrine disruption potential of pharmaceuticals and its consequences in reproduction after long term exposure

60 What are safe levels for endocrine disruptors in the environment?

61 What are the "hot spots" for studying retrospective PPCP effects to aquatic and terrestrial wildlife in developed and developing countries?

62 What are the interactions between ionisable PPCPs and counter ions within a waste water treatment system, and how might this interaction influence fate and exposure?

63 What are the key environmental exposure pathways for organisms (including humans) to PPCPs in the environment and are we missing any of these in current risk assessment approaches?

64 What can be done to harmonize evaluation of risk to aquatic or terrestrial organisms from exposure to pharmaceutical residues?

65 What contribution do antimicrobials in the environment make to the selection of resistant viruses, bacteria and fungi?

66 What factors affect the bioaccumulation and bioconcentration of pharmaceuticals in aquatic and terrestrial systems and food chains?

67 What impact do they have on aquatic ecosystems, especially fish?

68 What is the bioavailability of pharmaceuticals that are tightly bound to soil and sediment particles and can changes in environmental conditions affect this?

69 What is the current understanding of whether major drug contraindications will correspond to mixture toxicity in aquatic and terrestrial ecosystems?

70 What is the effect of sludge and manure-associated PPCPs on soil ecosystems?

71 What is the effort done to evaluate the level of pharmaceuticals and personal care product in indoor environment?

72 What is the fate of pharmaceuticals and what is the sensitivity of ecosystems and its components?

73 What is the level of exposure of organisms to pharmaceutical and personal care products in the Australian environment? Are these compounds accumulating in the environment?

74 What is the relative hazard and risk of pharmaceuticals compared to other chemicals?

75 What is the therapeutic dose of a drug to a 30 g fish, or a threatened or endangered unionid mussel?

76 What makes pharmaceuticals different to other chemicals?

77 What needs to be done to extend current environmental risk assessment schemes for Europe and N. America to other geographical regions with different emission scenarios and cultural practices?

78 What use can we have of genomic tools for the work in pharmaceutical impacts on the environment?

79 What will be the future environmental exposure to nano-pharmaceuticals and what will be the implications for this in terms of environmental risk?

80 What would an environmental versus socio-economic benefit analysis look like for a human medicine? Agreed case studies (hypothetic or real)?

81 What would be the impact(s) to the ecosystem health (as well as to individual organisms) from exposure to very low levels of contaminants on ongoing bases)?

82 What would be the socio-economic consequences of restricting pharmaceutical use based solely on categorization schemes highlighting adverse environmental hazard or risk?

83 When is the total residue approach insufficient for ERA?

84 Why can't there be an organized research effort to use pharmaceuticals and veterinary products as molecular probes to look for homologous responses in other species to expand our basic understanding of how organisms respond to well understood chemicals?

85 Why doesn't SETAC and other organizations outwardly support SMARxT Disposal and other trademark efforts to reduce PPCPs in the environment?

86 How can we assess risk of organic extract oil as a cosmetic and pesticide component?

87 Are antibiotic residues in the environment, especially from agriculture and aquaculture, contributing to the development of resistance in wildlife, and is there evidence that this could affect human health?

88 Drugs will not equally provoke biological effects on a per kg basis; can the DDD (as established by the WHO) serve as a proxy for the probability of inducing an effect? We could test the null hypothesis with algae and daphnia using fixed concentrations of a suite of drugs with different DDD values.

89 Environmental regulations for drugs are based on a threshold of effect. There is a need for an independent and non-industry sponsored review of the supporting data and review this approach in light of "non-threshold" concerns like carcinogenicity, endocrine effects, and antimicrobial resistance.

90 How are drugs from agricultural use actually entering the environment; that is, what proportion is direct liquid run-off to surface or ground water rather than "leachate from manure" applied to agricultural land?

91 How do terrestrial and aquatic ecosystem changes directly or indirectly affect human health? What are the aggregate effects on ecosystems of current-use and emerging toxicants? What are reliable scientific metrics for detecting chronic, long-term changes in ecosystems?

92 Do we need to be concerned about drug routes of exposure other than dietary for non-target terrestrial animals?

93 Effects data for non-target species are lacking for many drug classes, so priorities are needed for testing. Prioritization cannot be based on exposure or persistence alone due to potency of some drugs. How then should testing be prioritized?

94 How can pesticide registration ecotoxicity data (e.g. from the US) be better leveraged/used for assessing risks of pesticides registered as veterinary drugs?

95 Some have suggested use of drug development data for target species (e.g. humans) to identify potentially sensitive species/endpoints in terms of eco effects, based on degree of structural conservation of molecular drug targets. Is this reasonable? How could one demonstrate a "proof of concept"?

96 What would an efficient approach be for "discovery" of potential impacts of drugs, not related to their therapeutic targets, on plants, invertebrates and vertebrates without testing everything?

97 How to derive water quality guidelines or trigger values for PPCPs?

98 What are the realistic approaches and methods for Ecological Risk Assessment of mixtures of PPCPS in the receiving environment

99 How can we measure mixture toxicity of PPCPs in the aquatic environment?

100 Are regulators giving as much emphasis on the potential for endocrine activity to pharmaceuticals and personal care products as they are to agrochemicals?

101 What are the "safe" levels for discharge of PPCPs to the environment and in drinking water? This is currently based mostly around detection limits but is this representative or can we manage with higher levels?

102 What are the impacts of continuous release of complex PPCP mixtures, which are largely below instrument detection limits, to the environment?

103 What are the sub-lethal effects of PPCPs and how important is this for management? eg, spread/development of antibiotic resistance, modification of gender dominance?

104 What is the risk of bioaccumulation and impacts of PPCPs, particularly in human target species?

105 What sublethal effects can be observed for aquatic populations and at ecosystem level after chronic exposure at low concentrations?

106 What physical-chemical characteristics distinguish PPCPs from other chemicals that results in greater or lesser concern compared to "general chemicals"?

107 Would the regulation of PPCPs benefit from more clear or adjusted jurisdictional boundaries regarding their approval and use in commerce relative to environmental safety?

108 How can we prioritize which pharmaceuticals pose a threat to the environment? What are the effects and risks we anticipate?

109 How large is the impact of antibiotic production and usage on resistance in environmental bacteria? On human pathogens?

110 How will chronic exposure to mixtures of APIs affect organisms? Will there be epigenetic effects? Will effects be multigenerational?

111 Risks to the environment will always need to be weighed against benefits for human health, but should we be aiming to replace certain compounds with other, less environmentally dangerous APIs?

112 Given that environmental exposure is most likely to be continuous and chronic what are the appropriate endpoints to be explored in aquatic vertebrates / invertebrates?

113 How appropriate is chemical analysis for measuring the presence of pharmaceuticals in the environment? Would biological monitoring be more appropriate?

114 Is it possible to get industry to share the wealth of data they have gathered over the years on the various pharmaceuticals that are likely to cause environmental concern? Are any moves being made in this direction?

115 How widespread is the problem with emissions from pharmaceutical production?

116 Is a decreased selection pressure for acquiring antibiotic resistance, i.e., lower emissions of antibiotic substances into the environment, likely to result in also a lower abundance of antibiotic resistance genes in environmental bacteria? (Or is the acquired resistance genes preserved?)

117 What are the major drivers for antibiotic co-resistance?

118 What are the long-term effects of chronic exposure to low levels of PPCPs released into the environment on aquatic organisms including amphibians?

119 How much of a PPCP in surface water is bad for the environment?

120 What occurs in the environment in an aquatic organism (i.e., fish) when exposed to mixtures of pharmaceuticals with regard to drug-drug interactions?

121 Can the effects of PPCPs be isolated in sublethal toxicity tests conducted on (marine) organisms using whole municipal wastewater effluent?

122 How can a waste discharge manager more effectively assess the potential for effects of the PPCPs he/she monitors, particularly considering we are discharging an effluent that contains many other chemicals as well?

123 What is the effectiveness of various different treatment technologies on the removal/destruction of PPCPs in municipal wastewaters, sludges and biosolids?

124 What is the relevance of the effects of PPCPs in biosolids applied to land, particularly in relation to the effects of all the other chemicals potentially found in biosolids?

125 What is the relevance of the effects of PPCPs in relation to the effects of other chemicals that any given organism is exposed to?

126 What is the status of analytical methodologies for PPCPs and what are the implications of comparing results from non-standardized methods?

127 How can quantum chemical methods and atomistic simulations play a role in understanding environmental toxicology on the molecular scale?

128 In which chemical forms do PPCPs exist in aqueous environments: metabolic by-products, environmental degradation products, or original formulations? How does pH affect their chemical structure?

129 What is the PPCP disposal effect on marine ecosystems?

130 What steps can be taken to ensure protection of fish and their habitat from release of endocrine disrupting chemicals and other toxicants into the environment prior to our fully understanding potential adverse consequences of such releases?

131 What is the half-life of pharmaceutical products with high efficacy in the environment?

132 What are the main criteria test guidelines should met to address the risk for aquatic and terrestrial organisms? Are the current valid OECD guidelines (mentioned in the guidance document) appropriate to address the risk from pharmaceuticals with special mode of actions?

133 Can a laboratory test be devised to measure (or help to predict) pharmaceutical degradation half life to a reasonable degree of accuracy?

134 Can genomic techniques be used to identify modes of action that would be significant in the aquatic environment?

135 Can so-called "bound residues" of pharmaceuticals be removed from sediments and/or sludges by any process that might realistically occur in the environment?

136 Is it demonstrable from first principles that human metabolites of pharmaceuticals will have a lower ecotoxicity than the parent compound?

137 What is the occurrence (based on adequately sampled concentrations) of PPCPs in the environment?

138 What is the effect of pharmaceuticals and mixtures of pharmaceuticals on the microbial community and the ability to deliver vital ecosystem services?

139 What about long term uptake of pharmaceuticals by crops irrigated by wastewater treated by activated sludge, sand filtration and chlorination in which such compounds exist? What the dangers are expected to be for human health and animals (example: clover, animals, milk, humans).

140 Which chemicals have the potential to cause adverse biological effects?

141 How do the quantifiable adverse ecological and biological effects of pharmaceuticals and personal care products compare with the known effects of other natural and synthetic contaminants in the environment?

142 How can we determine the regulatory-level risk of these compounds in an efficient, timely manner?

143 What methods can be used for removal of cyanobacterial toxins?

144 What quantities of unused pharmaceuticals with unknown potential impacts on the environment are potentially in the community?

145 What are the effects to aquatic and terrestrial organisms from the chronic exposure to complex mixtures of PPCP?

146 Given that most PPCPs are discharged primarily via the use and disposal of domestic use, have there been suitable site-selection criteria been developed to ascertain the true potency of these materials in receiving water communities?

147 The concentration of various PPCPs has required an understanding of MoA's, especially in low concentrations. Have these chemicals been truly assessed for MoA that is relevant to receiving water communities?

148 Is the contribution of physiologist/biochemists on subtle/specific/low concentrations effects of pharmaceuticals on environmental fauna considered of some importance for guidelines definition, risk assessment, etc…?

149 Pharmaceuticals are specifically designed to induce specific effects at very low concentrations. How these subtle / low-dose / highly specific effects by single environmental pharmaceutical or their mixtures can be taken into account for guidelines development?

150 Increased information about the degradation patterns of these substances, the occurrence, persistence and ecotoxicity of degradation products as well as analytical techniques to detect them.

151 Many PPCPs are polar and ionizable substances to which the common models for partitioning and adsorption are not applicable. Part. and ads. are strongly related to bioavailability, and a qualitative as well as quantitative description of these mechanisms would greatly increase understanding.

152 Further research on mixtures. What are the potential mixture effects, what is the impact of mode or mechanism of action of individual compounds on any mixture effect, what are the dose response kinetics for mixture effects?

153 The presence of PhACs in the environment in high ng/L concentrations is pretty clear. What is unclear is what a safe concentration is? Access to pharmaceutical company's tox data would greatly improve our understanding of that.

154 What cost-effective technologies and other risk management strategies are best to reduce environmental releases of PPCPs from a variety of sources (e.g., from wastewater treatment plants, drinking water plants, concentrated animal feeding operations) that are adversely affecting ecosystems?

155 How to test for the effects of long-term low level exposure of humans to pharmaceuticals in the environment?

156 What are the major pathways of exposure of the major pharmaceuticals found in municipal effluent to the aquatic fauna and flora

157 What is the long-term toxicity of low concentrations of pharmacological mixtures to aquatic life?

158 Which trophic levels are at risk to a given class of pharmaceuticals?

159 What are the spatio-temporal cycles in ecosystem response to constant exposure of PPCPs with respect to its ecosystem services and how can they be actively managed to sustain an environment capable of enduring these exposures.

160 Will a regulatory framework be created that considers not only toxicological endpoints but concentration target, environmental loadings and presence of the substance in potable water after pharmaceuticals are released from domestic waste water streams?

161 What are differences in the fate and effects of the enantiomers of chiral PPCPs?

162 Are threshold limits proposed in the new Municipal Wastewater regulations protective of the aquatic receiving environment?

163 Does advanced wastewater treatment remove priority trace contaminants of concern to levels below which effects in the receiving environment are not expected?

164 How do researchers separate effects from PPCPs and other key stressors in this system such as ammonia, and water temperature on aquatic biota?

165 How do reproductive impacts demonstrated in a fish population exposed to treated sewage affect higher levels of biological organization (fish community, ecosystem)?

166 What are the links between estrogens or anti-androgens and reproductive dysfunction in the fish collected downstream of treated sewage discharges?

167 What are the most environmentally friendly methods to address the issue of veterinary and human pharmaceuticals in biosolids?

168 What are the threshold levels PPCPs which elicit a response in aquatic biota?

169 What is the fate, persistence and distribution of PPCPs in land applied biosolids?

170 What is the relative contribution of hormones derived from livestock operations compared to human waste streams to the receiving environment?

171 What is the relative contribution of PPCPs to natural hormones from human sources to effects in the receiving environment?

172 What is the relative contribution of veterinary drugs to PPCPs from human sources in the receiving environment?

173 impacts or effects in all environmental compartments (in terms of detected concentrations) and in all biota

174 Do observed concentrations of PPCPs translate into specific or aggregate effects of PPCPs on individual organisms, and does this in turn translate to effects on populations of aquatic organisms and/or ecosystems or ecosystem function and services

175 Are there any health effects from trace levels of PPCPs in drinking water, acting either alone or in combination?

176 Are the actions of pharmaceuticals conserved across different groups of organism?

177 How are the actions of pharmaceuticals affected by other stressors in the environment?

178 How do different classes of pharmaceuticals affect target species?

179 How does the interaction of a pharmaceutical with its receptor translate to an individual or population level effect?

180 What are the trans-generational impacts of exposures to pharmaceuticals?

181 Appreciation of chronic AOPs in non target organisms and linkage between mammalian and aquatic species toxicity responses and predictability based hereon

182 At what environmentally relevant concentration are PPCPs causing adverse effects to aquatic organisms that can negatively affect populations and communities of these organisms?

183 How can the variability (both environmental and anthropogenic) in emissions and exposure be more readily quantified to achieve a more realistic representation of spatial and temporal environmental exposure for regulatory decision making?

184 How does one calculate exposure risk for pharmaceutical mixtures in drinking water and integrate this input with possible interaction(s) with other environmental contaminants.

185 Do PPCP behave in the environment (e.g., transport and fate, toxicity) as predicted by SARs based primarily on industrial chemicals?

186 Can antibiotic resistant bacteria found in the environment be a potential threat to the human health care?

187 To what extent does the personal care products affect human life cycle(in particular fertility)?

188 Can we make more out of the available data if data transparency is increased and the data is coordinated more effectively?

189 Does ERA and ecopharmacovigilance need to consider off-label used of drugs?

190 How can we predict which pharmaceuticals are most likely to pose a risk?

191 Is ecopharmacovigilance a useful concept? If so, what would this mean in practice?

192 What are the risks associated with long-term exposure to pharmaceuticals at low levels on ecosystems and human health based on current and projected environmental concentrations, and how can they be assessed?

193 What makes an ERA fit-for-purpose?

194 How do we develop an integrated approach to effects based prioritization of pharmaceuticals in the environment? How do we ensure that the approach is balanced and inclusive of all active pharmaceutical ingredients?

195 Pharmaceuticals are biologically active compounds with specific modes of action. How do we integrate MOA based evaluations of aquatic species into risk assessments and eventually into the regulatory framework?

196 How do these biologically active chemicals impact or alter the functioning of food webs, either soil, terrestrial, or aquatic?

197 Do environmental levels of antibiotics induce/select for/have an impact on the prevalence of antibiotic resistance genes?

198 How can we resolve the effects of low concentrations of pharmaceuticals from the effects of the multiple other co-contaminants that are commonly encountered in real-life exposure scenarios?

199 How do we measure (biological/ecological)impacts of pharmaceuticals & personal care products (gene - individual - population)

200 Classifications of PPCPs by expected mode of action to test interactions?

201 How do the many changes in molecular endpoints translate into population health?

202 Interaction between PPCPs?

203 Can we identify biomarkers that will link specific chemicals or chemical classes directly to population or community-level impacts in aquatic ecosystems?

204 Based on current knowledge, what are the effects/risks of highest concern (human and environmental) related to emissions of pharmaceuticals?

205 How can economical incentives be created for industries to reduce environmental emissions from manufacturing?

206 How can the recent fast development in next generation sequencing and related technologies best help research on PPCPs in the environment?

207 How can we assess the risks that a pharmaceutical entering the external environment promotes resistance mechanisms, of a type that would be clinically relevant, and that these mechanisms then spread to the human microflora and eventually pathogens?

208 How can we best take advantage of the vast information on pharmaceuticals (kinetics and dynamics) generated from studies on mammals, including man?

209 How should we best incorporate sublethal responses of drugs in organisms in laboratory tests into the greater picture of environmental risk assessment?

210 Since antibiotic resistance, once developed, tend to spread all over the world, should we put most focus on managing the worst environmental antibiotic pollution sources regardless of where they are located, or should each country focus on their own backyard?

211 What are the (probably rather few?) pharmaceuticals that indeed have adverse environmental effects as a result of (normally low-level) emissions from usage?

212 What are the direct emissions of active pharmaceutical ingredients from manufacturing sites world-wide (concentrations, volumes) and what are the risks (human health and ecological) associated with such releases?

213 What are the possibilities and limitations in predicting effects of pharmaceuticals on wildlife based on similarities and differences between (the genomes of) species?

214 What are the risks for antibiotic resistance to develop in human pathogens as a result of environmental emissions of antibiotics at levels higher than human therapeutic levels(as found in industrial effluents from for example India, China and Korea)

215 What effluent treatment methods are effective in reducing the APIs causing the highest environmental risks, at the same time 1) not increasing the toxicity of the whole effluents, 2) being economically feasible, and 3) also being able to remove other unwanted organic contaminants?

216 What factors are most important for determining the flow of antibiotic resistance genes from environmental bacteria to human pathogens?

217 How do we determine (and convince the public that there are) environmental concentrations of PPCPs that are NOT of concern to organisms in lakes & rivers?

218 How do we link biomarker "screens" (YES, YAR etc) of MWWE or individual PPCP effects to real effects in the environment?

219 Risk Assessment of PPCPs is done chemical by chemical (or at best - by chemical class with similar MOA). How do we relate this to the MIXTURE of PPCPs in municipal wastewater effluents and in the environment?

220 Which pharmaceuticals and personal care products should we worry about? Which ones cause effects in the environment?

221 What are the high-risk environments for transfer of antibiotic resistance genes to human pathogens?

222 What are the risks that the promotion of antibiotic resistance genes seen in highly antibiotic-polluted environments will spread to pathogenic bacteria, and how does this affect our ability to fight bacterial infections?

223 How do specific sublethal effects and biomarkers of P&PCPs translate to ecological effects?

224 Risk assessment & prioritisation of P&PCPs in aquatic and soil ecosystems - which are the highest priority chemicals and what are their risks to the environment?

225 What is the epidemiological evidence that any PPCP chemicals are linked to disease in humans or wildlife?

226 Is it reasonable to assume that human metabolites as well as aerobic environmental TPs will have a lower inherent toxicity than parent compound? Is it possible to establish a risk quotient (RQ) below which the above metabolites and TPs may be dismissed without further consideration?

227 Can you correlate the harmful effects of PPCPs to their structural characteristics?

228 For pharmaceuticals: How can information generated during the drug development process on subjects such as mode of action, ADME (adsorption, distribution, metabolism, excretion), efficacy, safety, etc. be applied in the evaluation of potential effects on ecological receptors?

229 What data or studies exist to show that simple acute toxicity tests provide sufficient information to assess potentially chronic exposures to PPCPs for the wide spectrum of PPCPs? What is the range of known Acute-to-Chronic Ratios (ACRs) and do these ACRs reflect different modes of action?

230 Is the climatic difference between north and south or between coastal and continental, humid and dry, e.g., for North America and Europe, significant for the environmental fate (and ecotoxicity) of PPCP? If so, do we need to integrate that difference in PEC or PNECs or not?

231 What are the necessary tests (ordered by importance for fate) and defaults for modelling the environmental fate in surface waters? How can partitioning, surface water biodegradation, photodegradation and advection be intelligently integrated, what compartmental defaults should be used for modelling?

232 Are concentrations of antibiotics in the environment potentially high enough to lead to increases in antibiotic resistance?

233 What are the fate and transport of these compounds in WWTPs?

234 How do mixtures of PPCP affect aquatic life?

235 Removal of PPCP from drinking water?

236 Removal of PPCP from wastewater?

237 Given the conservation of drug targets between humans and other species, how do we take into account the potential for non-traditional effects (e.g. behavioural) associated with exposure to pharmaceuticals designed to perturb those metrics?

238 What is the ecological relevance of environmentally relevant concentrations

239 Do pharmaceuticals in the environment result in adverse effects and can we live with these effects?

240 What role do pharmaceutical metabolites play in the risk posed to the environment from PIE?

241 What is the impact of dissimilarly acting mixtures of pharmaceuticals in the environment?

242 Are CAFO's a significant part of the problem of release of PPCP's to aquatic environments, and if so, will they be required to treat effluent?

243 Are there multi-generational impacts of low, chronic doses of PPCP mixtures to aquatic and amphibious life?

244 How do impacts of PPCP's at the molecular and cellular level intersect or support observations at the environmental or community level?

245 How would phasing out the use of antibiotics in CAFO's, except in obviously sick animals, change the impact of effluent on aquatic systems?

246 If waste-water treatment plants are required to implement PPCP degradation technologies, will all plants be required to install them? If not, what criteria will be used to determine which plants install them, and who will develop the criteria and make the decisions?

247 What interactions do specific or multiple PPCP's have at the molecular level, particularly with respect to reproductive effects in aquatic life?

248 What is the potential economic impact of applying PPCP treatment technologies to large waste-water treatment plants, and what developing technologies could potentially decrease the economic impact?

249 How can we group pharmaceuticals and study them using representatives within an MOA to illustrate the effect of mixtures of pharmaceuticals within and across MOA on Aquatic and Terrestrial Organisms?

250 What are the various exposure profiles in freshwater and terrestrial ecosystems associated with sources of pharmaceuticals both human and animal?

251 What endpoints both traditional as well as molecular and behavioural can be used to identify MOA?

252 May alterations potentially resulting from climate changes modify substantially the toxicity, mechanisms of toxicity and pathways of biotransformation of these products?

253 What are the long-term effects of exposure to low concentrations of pharmaceuticals and personal care products, including complex mixtures, on populations of marine, especially estuaries?

254 What are the toxicological interactions between these substances and other environmental contaminants?

255 How widespread is the environmental pollution from pharmaceutical production and what is the local and global environmental impact?

256 To what extent will emission of antibiotics to the environment (through usage or production) provoke development of resistance that will have a significant impact on our health care? What is required for antibiotic resistance developed in the environment to spread to human pathogens?

257 What sewage treatment technologies are the most cost effective and best from an environmental perspective? How can we create economical incentive for the pharmaceutical industry to take care of the pollution from the production?

258 What is the relative contribution of the veterinary drugs and what of this is not for the treatment of animal safety issues? Are there alternatives for growth promotion?

259 What is (are) the effect(s) of multiple stressors in the receiving ecosystems?

260 What is the most suitable way to engage the communities in this issue?

261 What the mechanistic effects of pharmaceutical mixtures?

262 What is the best prioritization strategy to determine how the research efforts should be directed to minimize the negative effects of PPCPs on human health and ecosystems?

263 Effects of estrogens have been documented in the field, whole lake additions and in laboratory studies. What are the cumulative effects of multiple MWWE discharges in a receiving environment and are reproductive effects masked by nutrient effects?

264 Intersex has been documented in a number of species. Do we know enough about the frequency of this condition in unexposed populations?

265 Is there really anything to worry about in municipal wastes other than nutrients?

266 Do mixtures of low concentration of PPPCs give more effect to aquatic organisms than addition of individual impact?

267 How to prioritize which of these compounds a POTW should monitor and regulate with respect to effects on the marine environment?

268 What are effective policies and/or changes to waste management which can be integrated into environmental risk assessment to reduce adverse impacts of pharmaceuticals and personal care products in the environment?

269 Can the levels of pharmaceuticals measured in the aquatic environment be linked to expected levels in aquatic organisms, i.e., what factors can be used to predict bioavailability in aquatic organisms?

270 Ecotoxicological responses of marine organisms to triclosan and methyl-triclosan?

271 Factors leading to the methylation of triclosan in the environment?

272 Occurrence of triclosan and methyl-triclosan in stormwater or industrial effluents?

273 How can water and waste water utilities be assured that they are removing Pharmaceuticals and Personal Care Products from the water which will be sold to our customers or discharged to streams? What types of treatment techniques or systems will break-down and remove these products?

274 Is fish intersex caused by pharmaceuticals?

275 How can we combine the need of pharmaceuticals to treat disease and the environmental protection?

276 Of those PPCPs which persist in the aquatic environment, which aquatic life act as robust sentinels of potential human health effects?

277 What would be the impact(s) to overall ecosystem health (as well as to individual organisms) from exposure to very low levels of contaminants on an ongoing basis?

278 Can ‘‘omics’ and other novel technologies be used to identify pharmaceuticals that might pose a risk to the environment? If so, how?

279 Do mobile genetic elements that encode resistance in the environment pass resistance on to clinically important species?

280 How can information on the toxicity (e.g. side effects) and pharmacology of a pharmaceutical in humans be used to provide information on potential ecotoxicological and ecological effects (and vice versa)?

281 How do non-standard effects endpoints that have been observed in organisms exposed to pharmaceuticals, such as biomarker responses, relate to ecologically important endpoints such as survival, growth and reproduction?

282 How important is metabolism for reducing bioconcentration in wildlife?

283 How important is the contribution of pharmaceuticals compared to other contaminants to real-world mixture toxicity?

284 How should pharmaceuticals be prioritised for assessment in terms of their impact on the environment?

285 To what extent do pharmaceuticals in the environment get into food supplies and what are the implications of this in terms of risks to human health?

286 What are the effects, if any, of bound residues of pharmaceuticals in soils and sediments on organisms?

287 What are the risks associated with long-term exposure to pharmaceuticals at low levels on ecosystems and human health based on current and projected environmental concentrations, and how can they be assessed?

288 What contribution do residues of antimicrobials in the environment make to the selection of resistant viruses, bacteria and fungi that are important for human and animal health?

289 What data which we currently collect are most valuable for risk assessment and what can be discarded?

290 What evidence is there that pharmaceuticals in the environment are affecting ecosystems and human health?

291 What factors affect the bioaccumulation and bioconcentration of pharmaceuticals in aquatic and terrestrial systems and food chains?

292 What is the level of risk of pharmaceuticals to wildlife (e.g. birds), ecosystem service provisioning and broader biodiversity?

293 What needs to be done to extend current environmental risk assessment schemes e.g. for Europe and N. America to other geographical regions?

294 What strategies exist to reduce environmental exposure and effects of pharmaceuticals when a potential risk is identified?

295 When do the standard risk assessment methodologies fail to identify the potential for adverse environmental effects?

296 Which catchments or regions are most at risk from exposure to pharmaceuticals?

297 How to create a general database for all the studies

298 How can we identify their adverse effects in complex mixtures like sewage effluents?

299 How similar are the occurrence patterns around the world (i.e. developed and developing countries)?

300 In contrast to ECD’s, PPCP’s are a heterogeneous group from the point of view of the biological effects they elicit. It is the common source enough as rationale justification to evaluate them all together?

301 There is any relationship among their clinical mechanism of action in humans and in wildlife?

302 There is any relationship among use and environmental occurrence patterns?

303 Which are the differences that distinguish PPCP’s from other traditional pollutants? Do we need to develop specific ecotoxicological methods of assessment?

304 Which are the environmental levels in relation with the sensitivity of local, especially considering highly biodiversity sites?

305 How might chemicals be grouped for evaluation?

306 How might chemicals be prioritized for environmental monitoring?

307 How might the adverse impact (risk) of one chemical exposure be measured against all other chemical exposures, and how might all chemical exposures be measured against all other stressors?

308 What is the effect of organism exposure to multiple PPCPs

309 Are wastewater treatment plant discharges, to water and/or irrigating crops, the only plausible route for significant build-up of pharmaceuticals and personal care products in the environment?

310 Is there a need for more studies investigating long-term, low-level effects of PPCPs in aquatic and terrestrial systems in support of assessing potential risks?

311 Why is there not more emphasis on communicating the risks of PPCPs to the public?

312 Understanding of the relative potencies of metabolically or environmentally modified molecules and the environmental chemical factors that can lead to "reactivation" of conjugated molecules.

313 Ecological relevance of pharmaceuticals in the environment: What methods can be employed to determine/measure in situ effects of individual APIs or mixtures of similar acting compounds? How predictive are the laboratory data produced for risk assessments?

314 Mechanism based testing strategy: The ecotoxicological testing of APIs for risk assessment is based mainly on standard procedures. How can we develop specific test methods, which appreciate the different modes of actions of APIs, for example as it is done for endocrine active compounds?

315 What happens to spent fermentation media? Fermentations are used to create many drug products. After fermentation is complete the drug is removed from the fermentation mass. What happens to the spent fermentation mass? Is it re-used, used for animal feed or discarded to the environment?

316 What is the environmental impact of chemicals in suncare products to marine environments? Many tons of suncare products are used each year and enter the marine environment what is their fate and do they negatively impact fish, plankton or living corals?

317 Beyond biomarker induction, we need consensus on criteria for assessing the magnitude of environmental effects and a suite of standardized lab tests with endpoints directly related to the environmental criteria.

318 Are pharmaceutical residues present in the environment contributing to the current decline in biodiversity?

319 Can biomarker responses be related to ecologically relevant population endpoints?

320 Can concentration thresholds of concern be used to address mixture risks?

321 Can current or new technologies or approaches be used to identify the most sensitive species for testing based on mode-of-action of a given pharmaceutical?

322 Can drug-drug interactions be used to identify potentially synergistic effects in pharmaceutical residue mixtures?

323 Can more accurate partitioning models be developed for pharmaceuticals so that environmental concentration estimates are more accurate?

324 Can threshold concentrations based on biological activity be determined that are generally protective of humans and environmental organisms?

325 Can wild populations be systematically sampled to determine in vivo concentrations of prioritized pharmaceuticals?

326 Does the presence of anti-infective pharmaceutical residues in the environment contribute to the development of resistance?

327 How can pharmaceutical preclinical and clinical information (ADMET) be used to assess the potential for adverse environmental impacts and customize subsequent testing strategies?

328 Is the current standardized testing regime and environmental risk assessment methodology for pharmaceuticals protective of both humans and environmental organisms?

329 Is there an agreed prioritization approach to assess pharmaceuticals based on their intrinsic hazards and potential risks to the environment?

330 Is there any evidence of increased environmental exposures to humans or organisms via concentration or accumulation effects in the terrestrial systems and associated food chains?

331 What are the effects of bound residues of pharmaceuticals and aging processes in soils and sediments?

332 What are the gaps in current pharmaceutical and ecological knowledge that need to be filled in order to assess potential human and ecosystem effects of long-term low level exposures to pharmaceuticals?

333 What human sensitive sub-populations or regional or local ecosystems would be at the most risk from exposure to pharmaceutical residues?

334 Will rising concentrations of pharmaceutical residues as a result of decreased water use and changing precipitation patterns put these watershed ecosystems at more risk?

335 What is the environmental fate of these types of substances?

336 What is the ecotoxicology of long chain (> C8) and sulfonated perflourinated chemicals?

337 What level of pharmaceuticals and personal care products will not impair water quality for aquatic life and human health?

338 What is the impact of PPCP from STPs in tropical marine environments

339 To what extent can the intended mode of action of pharmaceuticals be used to predict likely impacts on ecosystems?

340 How do pharmaceuticals impact trophic levels and are there synergistic effects?

341 Since there is evidence that P&PC actives are getting into wastewater are there economical technologies that can be added to waste treatment facilities that can inactivate these actives?

342 Are any excipients of environmental relevance?

343 Are appropriate disposal systems for pharmaceuticals available?

344 Can hygiene management in stables and changes in animal husbandry reduce the amount of veterinary pharmaceuticals used? How can such good management practices be implemented bindingly?

345 Do antibiotics have indirect negative effects in the environment by selecting certain microbial strains?

346 Do emissions from production sites of pharmaceuticals pose a risk to the environment? Is additional regulation needed (in the EU, North America, in Asia)? If yes, what would be an appropriate regulation?

347 Do human and veterinary pharmaceuticals in the environment pose a risk to humans? Does the current risk for humans differ between regions?

348 Do the OECD guidelines currently used for the environmental risk assessment of human and veterinary pharmaceuticals cover the appropriate organisms? Would it be necessary to add molluscs to the testing program?

349 How can an environmental risk assessment be conducted for pharmaceutical active ingredients that are no standard small molecule drugs (e.g. biopharmaceuticals and nanomedicines)?

350 How can doctors, veterinarians, farmers, pharmacists, etc. be educated about the risk human and veterinary pharmaceuticals pose to the environment?

351 How can transformation products of active ingredients of human and veterinary pharmaceuticals be assessed?

352 How could all information on occurrence, fate, and effects of human and veterinary pharmaceuticals be summarized and made available to stakeholders and the interested public (maps with all concentrations of detected pharmaceuticals? ERA-wiki?)

353 How could duplicate testing of active ingredients of human and veterinary pharmaceuticals be avoided?

354 How could effects of pharmaceuticals in the environment be detected within the pharmacovigilance system?

355 How could measures to prevent and cure illnesses without the use of pharmaceuticals be promoted?

356 How could monitoring be used to evaluate the effectiveness of environmental risk assessments for human and veterinary pharmaceuticals?

357 How could non-lipid based mechanisms of accumulation of active ingredients into organisms be taken into account for the environmental risk assessment of human and veterinary pharmaceuticals?

358 How could the risk for the environment be taken into account in the benefit-risk-assessment for veterinary pharmaceuticals?

359 How do human and veterinary pharmaceuticals affect biodiversity and protected species? Are those effects covered by the current risk assessment?

360 How should a monitoring scheme for authorized pharmaceuticals which potentially pose a risk to the environment be designed?

361 How should environmental concentrations of human and veterinary pharmaceuticals be predicted (PEC calculation) in order not to underestimate the concentration in the environment (no PECs lower than MECs)?

362 How should genotoxicity of an active ingredient be assessed with regard to environmental organisms?

363 How should non-extractable residues of human and veterinary pharmaceuticals be assessed? How could climatic variability (seasonal as well as long-term) be taken into account in the assessment of NER?

364 How should PBT pharmaceuticals be managed?

365 Is a limit of 0.1 µg/l for human and veterinary pharmaceuticals in ground water protective for the ecosystem and humans?

366 Is it a realistic assumption that green pharmacy will reduce the risk human and veterinary pharmaceuticals pose to the environment in the coming years, e. g. will actives that easily mineralize be developed?

367 Is it acceptable that human and veterinary pharmaceuticals enter ground and drinking water?

368 Is it appropriate to trigger an experimental environmental risk assessment based on a threshold predicted environmental concentration? If yes, which trigger value would be appropriate?

369 Is there a correlation between dose and effect values in the environment? Do pharmaceuticals which are used at lower doses show effects at lower concentrations? If yes, which consequences does this have for the use of trigger values and the prediction of environmental concentrations?

370 Should any non-standard endpoints be included in the environmental risk assessment of pharmaceuticals?

371 Should climatic differences be taken into account in the environmental risk assessment of human and veterinary pharmaceuticals (within the EU and between regions)?

372 Should pharmaceuticals be classified and labelled (GHS)?

373 What are the benefits and risks of nano-pharmaceuticals for the environment?

374 What effects does the use of wild plants or animals as pharmaceuticals have on the environment/biodiversity? If there are adverse effects, would additional regulation be needed?

375 What is the correct disposal pathway for human and veterinary pharmaceuticals to avoid entry into the environment? Are there differences between regions?

376 What would be an appropriate approach to assess mixture toxicity in the ERA for human and veterinary pharmaceuticals?

377 What would be an appropriate test for inhibition of microbial activity in the environment (besides STPs) by pharmaceuticals?

378 What would be effective risk mitigation measures for human and veterinary pharmaceuticals?

379 What would be the best approach to take the use of an active substance in several products into account for exposure assessment for human and veterinary pharmaceuticals? How can the use of this active for other purposes (e. g. as pesticide) be taken into account?

380 What would be the best way to assess the environmental risk of existing human and veterinary pharmaceuticals?

381 Which additional environmental standards should be implemented for the production of human and veterinary pharmaceuticals?

382 Which effect does the price of pharmaceuticals and the health care system have on the entry of human and veterinary pharmaceuticals into the environment?

383 Which options for action do different professionals (doctors, veterinarians, farmers, pharmacists, drinking water providers, sewage treatment plant operators, pharmaceutical industry, patients, etc.) have to reduce the environmental risk of pharmaceuticals?

384 Would an ecolabel be a successful approach to reduce the environmental risk of human and veterinary pharmaceuticals?

385 Would an environmental classification system for human and veterinary pharmaceuticals be helpful to reduce the environmental risk of pharmaceuticals? What is the best approach? Should it be a local system or harmonized between different regions? Which stakeholders would have to be approached?

386 Would it be acceptable that authorization of a human pharmaceutical is denied based on the risk for the environment? Would it be possible to restrict authorization only to patient groups or indications for which no appropriate alternative treatment option is available?

387 Would it be appropriate to harmonize the environmental risk assessment for human and veterinary pharmaceuticals on a global scale?

388 Would it be feasible to take into account the environmental risk of human pharmaceuticals during benefit-risk-analysis?

389 What are "real" hazard concentration limits (contamination vs. pollution), and does the use and help of such products equal an environmental risk?

390 endocrine disrupting effects of chemicals reproductive effects adverse health and environmental effects of nanoforms partitioning data to identify quasi persistence

391 To what extent can non target species be more affected than targets and consequences for the functioning of the ecosystem

392 To what extent will the emission of antibiotic substances to the environment by drug use or production activities provoke the development of resistance that will have a significant impact on our combat of infections?

393 Is the significance of the impacts of pharmaceuticals/PCPs the same for human health as it is for ecosystems? I.e., should we assess risks similarly for both?

394 Monitoring of disposal of pharmaceuticals and personal care products- what measures are we taking to educate the public and what options are we providing to the public to be compliant for disposing these items

395 Which are the biochemical, biological (natural science), technological and institutional (social science) prerequisites and limitations for an innovative re-engineering of PPCPs to easily (bio) degradable substances?

396 How to identify the effects of PPCPs with low dose (and long-term) exposure?

397 A database of concentrations of pharmaceuticals and personal care products in sewage effluent, grey water etc would be really useful.

398 Can we develop a removal system to eliminate the majority of the structurally diverse pharmaceuticals from sewage effluent?

399 What are the bioconcentration and bioaccumulation potentials of selected model compounds?

400 Mixture effects of PCP and occurrence of PCP?

401 Given the considerable benefits from pharmaceuticals and personal care products, is it really necessary to manage the risks to ecosystems? Are they almost negligible?

402 What really are the field effects on ecosystems?

403 What level will these PPCPs get into drinking source water? How would these PPCPs at their current environmental exposure level adversely affect aquatic organisms at critical life stage?

3. Questions taken forward to the workshop (broken down by breakout group)

BG1. Ecological effects of PPCP exposure (12)

1 What evidence is there that pharmaceuticals in the environment are affecting ecosystems and human health?

2 Are herbivorous, omnivorous and piscivorous birds and other wildlife at risk from PPCP exposures?

3 How do human and veterinary pharmaceuticals affect biodiversity and protected species? Are those effects covered by the current risk assessment?

4 What is the effect of sludge and manure-associated PPCPs on soil ecosystems?

5 What is the effect of pharmaceuticals and mixtures of pharmaceuticals on the microbial community and the ability to deliver vital ecosystem services?

6 What are the spatio-temporal cycles in ecosystem response to constant exposure of PPCPs with respect to ecosystem services and how can they be actively managed to sustain an environment capable of enduring these exposures.

7 Which trophic levels are at risk to a given class of pharmaceuticals?

8 How do non-standard effects endpoints that have been observed in organisms exposed to pharmaceuticals, such as biomarker responses, relate to ecologically important endpoints such as survival, growth and reproduction?

9 What is the environmental impact of chemicals in suncare products to marine environments? Many tons of suncare products are used each year and enter the marine environment what is their fate and do they negatively impact fish, plankton or living corals?

10 Ecotoxicological responses of marine organisms to triclosan and methyl-triclosan?

11 What effects does the use of wild plants or animals as pharmaceuticals have on the environment/biodiversity? If there are adverse effects, would additional regulation be needed?

12 What is (are) the effect(s) of multiple stressors in the receiving ecosystems?

BG2. Use of safety data and mode of action data (15)

13 How can pharmaceutical preclinical and clinical information (ADMET, side effect data, drug-drug interactions etc) be used to assess the potential for adverse environmental impacts of pharmaceuticals and pharmaceutical mixtures and customize subsequent testing strategies?

14 If the proposed approaches to leverage mammalian or target organism pharmacology and toxicology data to predict aquatic and terrestrial wildlife effects of PPCPs are promising, what data are needed to experimentally validate these potentially predictive models?

15 Can ''omics' and other novel technologies be used to identify pharmaceuticals and pharmaceutical modes of action that might pose a risk to the environment? If so, how?

16 Is there a correlation between dose and effect values in the environment? Do pharmaceuticals which are used at lower doses show effects at lower concentrations? If yes, which consequences does this have for the use of trigger values and the prediction of environmental concentrations?

17 Are the actions of pharmaceuticals conserved across different groups of organism?

18 Some have suggested use of drug development data for target species (e.g. humans) to identify potentially sensitive species/endpoints in terms of eco effects, based on degree of structural conservation of molecular drug targets. Is this reasonable? How could one demonstrate a "proof of concept"?

19 What endpoints both traditional as well as molecular and behavioural can be used to identify MOA?

20 Mechanism based testing strategy: The ecotoxicological testing of APIs for risk assessment is based mainly on standard procedures. How can we develop specific test methods, which appreciate the different modes of actions of APIs, for example as it is done for endocrine active compounds?

21 Why can't there be an organized research effort to use pharmaceuticals and veterinary products as molecular probes to look for homologous responses in other species to expand our basic understanding of how organisms respond to well understood chemicals?

22 Do PPCP behave in the environment (e.g., transport and fate, toxicity) as predicted by SARs based primarily on industrial chemicals?

23 Can you correlate the harmful effects of PPCPs to their structural characteristics?

24 How can we group pharmaceuticals and study them using representatives within an MOA to illustrate the effect of mixtures of pharmaceuticals within and across MOA on Aquatic and Terrestrial Organisms?

25 Pharmaceuticals are biologically active compounds with specific modes of action. How do we integrate MOA based evaluations of aquatic species into risk assessments and eventually into the regulatory framework?

26 The concentration of various PPCPs has required an understanding of MoA's, especially in low concentrations. Have these chemicals been truly assessed for MoA that is relevant to receiving water communities?

27 How does the interaction of a pharmaceutical with its receptor translate to an individual or population level effect?

BG3. Bioavailability, uptake and metabolism (11)

28 How can the BCF and BAF of ionisable organics be measured or modelled?

29 Do ADME characteristics in mammals predict ADME of pharmaceuticals in fish models? For example, are Vd, Cmax, t1/2, Cl values from humans predictive of those parameters in a 700 g fish or a red-eared slider turtle?

30 What is the bioavailability of pharmaceuticals to aquatic and terrestrial organisms, including those that are tightly bound to soil and sediment particles, and can changes in environmental conditions affect this?

31 What factors affect the bioaccumulation and bioconcentration of pharmaceuticals in aquatic and terrestrial systems and food chains?

32 What is the risk of bioaccumulation and impacts of PPCPs, particularly in human target species?

33 How can transformation products of active ingredients of human and veterinary pharmaceuticals be assessed?

34 Is it demonstrable from first principles that human metabolites of pharmaceuticals will have a lower ecotoxicity than the parent compound?

35 What role do pharmaceutical metabolites play in the risk posed to the environment from PIE?

36 Should regulatory assessments account for the influences of site specific pH on bioavailability and effects of weak acid and weak base PPCPs?

37 Should regulatory assessments of PPCPs account for differences in metabolism and effects of enantiomers? E.g., Why is chirality not accounted for in PPCP ERAs?

38 Is it appropriate to use Kow for ionisable organics, or would Dow be more appropriate as a trigger value within a regulatory context?

BG4. Antibiotic resistance and human health (8)

39 Does environmental exposure to antimicrobial residues, or to antimicrobial-resistance determinants selected for anthropogenically, promote resistance in environmental microorganisms, and create a reservoir that is then important in resistance development in human pathogens or commensals and what factors affect this?

40 Does resistance pass from the natural environment to the clinical environment and result in hospital acquired infections?

41 Is a decreased selection pressure for acquiring antibiotic resistance, i.e., lower emissions of antibiotic substances into the environment, likely to result in also a lower abundance of antibiotic resistance genes in environmental bacteria? (Or is the acquired resistance genes preserved?)

42 How can we assess the risks that a pharmaceutical entering the external environment promotes resistance mechanisms, of a type that would be clinically relevant, and that these mechanisms then spread to the human microflora and eventually pathogens?

43 Since antibiotic resistance, once developed, tend to spread all over the world, should we put most focus on managing the worst environmental antibiotic pollution sources regardless of where they are located, or should each country focus on their own backyard?

44 To what extent do pharmaceuticals in the environment get into food supplies and what are the implications of this in terms of risks to human health?

45 Are there any health effects from trace levels of PPCPs in drinking water, acting either alone or in combination?

46 To what extent does the personal care products affect human life cycle (in particular fertility)?

BG5. Regulatory risk assessment and testing (14)

47 Should medicines with both human and veterinary applications be assessed within a single framework to capture the ‘collective’ risk?

48 Should we collect the same amount of human health and environmental data on personal care products, and especially "natural" products, as we do for pharmaceuticals?

49 What are appropriate risk assessment processes for whole effluent and mixtures? Should the chemical-by-chemical assessment approach be abandon or does it still have an appropriate place in an assessment paradigm?

50 How can we determine the regulatory-level risk of these compounds in an efficient, timely manner?

51 What data which we currently collect are most valuable for risk assessment and what can be discarded?

52 Should any non-standard endpoints be included in the environmental risk assessment of pharmaceuticals?

53 What can be done to harmonize evaluation of risk to aquatic or terrestrial organisms from exposure to pharmaceutical residues?

54 Is the current standardized testing regime and environmental risk assessment methodology for pharmaceuticals protective of both humans and environmental organisms?

55 Is the terrestrial compartment sufficiently protected by current ERA procedures?

56 What would be an appropriate test for inhibition of microbial activity in the environment (besides STPs) by pharmaceuticals?

57 How should genotoxicity of an active ingredient be assessed with regard to environmental organisms?

58 How can an environmental risk assessment be conducted for pharmaceutical active ingredients that are no standard small molecule drugs (e.g. biopharmaceuticals and nanomedicines)?

59 Are cladoceran reproduction assays appropriate for characterizing chronic toxicity of all PPCPs?

60 What would be an appropriate approach to assess mixture toxicity in the ERA for human and veterinary pharmaceuticals?

BG6. Risk management (8)

61 If a drug has an adverse PBT and/ or environmental risk profile what can be done to manage and mitigate the risks? Case studies?

62 Is green drug design feasible? How will it work? And, what are the success criteria or protection goals? What if greener drug design is not feasible?

63 Would an environmental classification system for human and veterinary pharmaceuticals be helpful to reduce the environmental risk of pharmaceuticals? What is the best approach? Should it be a local system or harmonized between different regions? Which stakeholders would have to be approached?

64 What effluent treatment methods are effective in reducing the APIs causing the highest environmental risks, at the same time 1) not increasing the toxicity of the whole effluents, 2) being economically feasible, and 3) also being able to remove other unwanted organic contaminants?

65 How would phasing out the use of antibiotics in CAFO's, except in obviously sick animals, change the impact of effluent on aquatic systems?

66 How could the risk for the environment be taken into account in the benefit-risk-assessment for veterinary pharmaceuticals?

67 What would be the socio-economic consequences of restricting pharmaceutical use based solely on categorization schemes highlighting adverse environmental hazard or risk?

68 How should we manage the risks associated with pharmaceuticals that have environmental data gaps?

BG7. Thresholds of concern and long-term exposure (11)

69 Can threshold concentrations based on biological activity be determined that are generally protective of humans and environmental organisms?

70 Environmental regulations for drugs are based on a threshold of effect. There is a need for an independent and non-industry sponsored review of the supporting data and review this approach in light of "non-threshold" concerns like carcinogenicity, endocrine effects, and antimicrobial resistance.

71 At what environmentally relevant concentration are PPCPs causing adverse effects to aquatic organisms that can negatively affect populations and communities of these organisms?

72 Is it possible to get industry to share the wealth of data they have gathered over the years on the various pharmaceuticals that are likely to cause environmental concern? Are any moves being made in this direction?

73 Can we make more out of the available data if data transparency is increased and the data is coordinated more effectively?

74 What are the risks associated with long-term exposure to pharmaceuticals at low levels on ecosystems and human health based on current and projected environmental concentrations, and how can they be assessed?

75 What is the long-term toxicity of low concentrations of pharmacological mixtures to aquatic and terrestrial life?

76 What data or studies exist to show that simple acute toxicity tests provide sufficient information to assess potentially chronic exposures to PPCPs for the wide spectrum of PPCPs? What is the range of known Acute-to-Chronic Ratios (ACRs) and do these ACRs reflect different modes of action?

77 Given that environmental exposure is most likely to be continuous and chronic what are the appropriate endpoints to be explored in aquatic vertebrates / invertebrates and humans?

78 What are the gaps in current pharmaceutical and ecological knowledge that need to be filled in order to assess potential human and ecosystem effects of long-term low level exposures to pharmaceuticals?

79 What are the trans-generational impacts of exposures to pharmaceuticals?

BG8. Prioritization and relative risk issues (11)

80 Which pharmaceuticals and personal care products should we worry about? Which ones cause effects in the environment?

81 What is the best prioritization strategy to determine how the research efforts should be directed to minimize the negative effects of PPCPs on human health and ecosystems?

82 What would an efficient approach be for "discovery" of potential impacts of drugs, not related to their therapeutic targets, on plants, invertebrates and vertebrates without testing everything?

83 How do different classes of pharmaceuticals affect target species?

84 What is the relative contribution of hormones derived from livestock operations compared to human waste streams to the receiving environment?

85 What is the relative contribution of veterinary drugs to PPCPs from human sources in the receiving environment?

86 How can we resolve the effects of low concentrations of pharmaceuticals from the effects of the multiple other co-contaminants that are commonly encountered in real-life exposure scenarios?

87 How do risks from trace levels of pharmaceutical and personal care products in the environment compare to other human and environmental risks facing society, and how can we quantify these risks for comparison?

88 What physical-chemical characteristics distinguish PPCPs from other chemicals that results in greater or lesser concern compared to "general chemicals"?

89 What is the relative contribution of PPCPs to natural hormones from human sources to effects in the receiving environment?

90 How important is the contribution of pharmaceuticals compared to other contaminants to real-world mixture toxicity?

BG9. Temporal and spatial variations in exposure and risk (10)

91 How will environmental change affect the use and fate of pharmaceuticals and personal care products and how will this affect environmental risks compared to today?

92 Will rising concentrations of pharmaceutical residues as a result of decreased water use and changing precipitation patterns put these watershed ecosystems at more risk?

93 What are the key environmental exposure pathways for organisms (including humans) to PPCPs in the environment and are we missing any of these in current risk assessment approaches?

94 Is the climatic difference between north and south or between coastal and continental, humid and dry, e.g., for North America and Europe, significant for the environmental fate (and ecotoxicity) of PPCP? If so, do we need to integrate that difference in PEC or PNECs or not?

95 What needs to be done to extend current environmental risk assessment schemes for Europe and N. America to other geographical regions with different emission scenarios and cultural practices?

96 How can we effectively target which areas or regions are most at risk to health issues related to pharmaceuticals in the environment? Are these risks related to manufacturing, use, population demographics or the presence or absence of infrastructure?

97 What are the direct emissions of active pharmaceutical ingredients from manufacturing sites world-wide (concentrations, volumes) and what are the risks (human health and ecological) associated with such releases? How can this be better regulated?

98 Can more accurate partitioning models be developed for pharmaceuticals so that environmental concentration estimates are more accurate?

99 What are the various exposure profiles in freshwater and terrestrial ecosystems associated with sources of pharmaceuticals both human and animal?

100 What is the effort done to evaluate the level of pharmaceuticals and personal care product in indoor environment?

101 In which chemical forms do PPCPs exist in aqueous environments: metabolic by-products, environmental degradation products, or original formulations? How does pH affect their chemical structure?

4. References

Fleishman E, Blockstein DE, Hall JA, Mascia MB, Rudd MA, Scott JM, et al. 2011. Top 40 priorities for science to inform conservation and management policy in the United States. Bioscience 61:290-300.

Rudd MA, Beazley K, Cooke SJ, Fleishman E, Lane DE, Mascia MB, et al. 2010. Generation of priority research questions to inform conservation policy and management at a national level. Conserv Biol 25:476-484

Sutherland WJ, Fleishman E, Mascia MB, Pretty J, Rudd MA. 2011. Methods for collaboratively identifying research priorities and emerging issues in science and policy. Method Ecol Evol 2:238-247.
